# Exogenous Ang-(1-7) inhibits autophagy via HIF-1α/THBS1/BECN1 axis to alleviate chronic intermittent hypoxia-enhanced airway remodelling of asthma

**DOI:** 10.1038/s41420-023-01662-0

**Published:** 2023-10-02

**Authors:** Jian Ping Zhou, Yi Wang, Shi Qi Li, Jia Qi Zhang, Ying Ni Lin, Xian Wen Sun, Li Na Zhou, Liu Zhang, Fang Ying Lu, Yong Jie Ding, Qing Yun Li

**Affiliations:** 1 Department of Respiratory and Critical Care Medicine, Ruijin Hospital, Shanghai Jiao Tong University School of Medicine, Shanghai, 200025 China; 2 Institute of Respiratory Diseases, Shanghai Jiao Tong University School of Medicine, Shanghai, 200025 China; 3 Shanghai Key Laboratory of Emergency Prevention, Diagnosis and Treatment of Respiratory Infectious Diseases, Shanghai, 200025 China; 4 Department of Pharmacy, Shanghai Municipal Hospital of Traditional Chinese Medicine, Shanghai University of Traditional Chinese Medicine, Shanghai, 200071 China

**Keywords:** Asthma, Macroautophagy

## Abstract

Obstructive sleep apnoea (OSA)-induced chronic intermittent hypoxia (CIH) has been considered a risk factor for severe asthma. Airway remodelling, which could be modulated by autophagy, plays a key role in severe asthma. However, the extent of autophagy’s involvement in CIH-potentiated airway remodelling remains largely unexplored. Furthermore, we had found that angiotensin-(1-7) [Ang-(1-7)] has therapeutic effects on airway remodelling in asthma, but the underlying mechanism is either unclear. This study aimed to explore how CIH aggravates asthma and mechanism of protective effects of Ang-(1-7) on airway remodelling, with a focus on autophagy. We observed that CIH promoted epithelial-to-mesenchymal transition (EMT), indicated by elevated EMT and fibrotic markers such as Snail and Collagen IV, both in vitro and in vivo. CIH intensified cell autophagy, evident from increased LC3B expression and reduced p62 levels. Ang-(1-7) reversed the CIH-enhanced expression of Snail, Collagen IV, and LC3B. To explore how CIH enhanced autophagy in cellular and animal model of asthma, overexpression of hypoxia-inducible factor 1-alpha (HIF-1α) and Thrombospondin 1 (THBS1) were identified in CIH-exposure mice lung compared with normal mice lung tissues from the GEO database. Finally, through chromatin immunoprecipitation and immunoprecipitation assays, we verified that Ang-(1-7) inhibits CIH-induced binding of HIF-1α to the promoter of THBS1, and also disrupts the protein-protein interaction between THBS1 and the autophagy-associated protein Beclin 1 (BECN1), ultimately leading to autophagy inhibition. Our findings suggest that exogenous Ang-(1-7) can inhibit autophagy via HIF-1α/THBS1/BECN1 axis, thereby alleviating CIH-enhanced airway remodelling in asthma. These findings imply the potential therapeutic effect of Ang-(1-7) in asthma with OSA.

## Introduction

Asthma is a common respiratory condition, and it is estimated that around 3.6 to 10% of asthmatic patients have difficult-to-control asthma, resulting in a poor quality of life [1]. Studies have suggested that refractory asthma can be exacerbated by obstructive sleep apnoea (OSA), with OSA being present in over 50% of patients with severe asthma [2, 3]. OSA is significantly associated with frequent asthma exacerbations [4]. OSA-related upper airway collapse and soft tissue damage can lead to bronchoconstriction, increasing the risk of nocturnal asthma [5]. Moreover, chronic intermittent hypoxia (CIH), the main pathophysiological process of OSA, causes bronchial hyperreactivity and thus gives rise to airway and systemic inflammation and consequently increases the risk of difficult-to-control asthma [6, 7].

Several studies have been conducted to investigate the role of the renin-angiotensin system (RAS), particularly octapeptide angiotensin II (Ang II), in the progression of airway remodelling [8]. In murine asthma models, elevated Ang II levels have been shown to directly contribute to airway smooth muscle cell hyperresponsiveness, thereby exacerbating airway remodelling [9–11]. Angiotensin-(1-7) [Ang-(1-7)], a counter-regulatory mediator of Ang II, is generated from the degradation of Ang II by angiotensin-converting enzyme 2 (ACE2). It interacts with the Mas receptor and exhibits anti-inflammatory and anti-fibrotic effects [12–14]. These findings suggested that dysregulated balance between Ang II and Ang-(1-7) could play a significant role in epithelial-to-mesenchymal transition (EMT) and airway remodelling. Furthermore, studies have revealed that CIH can upregulate the local RAS, while Ang-(1-7) shows potential to mitigate lung injury by inhibiting inflammation and oxidative stress [15–17]. Our previous investigation has confirmed that Ang-(1–7) ameliorates CIH-aggravated airway remodelling in murine and cellular models of asthma. However, the precise molecular mechanisms underlying this effect have yet to be fully understood.

Autophagy has been extensively investigated in various pulmonary contexts, and its excessive activation has been associated with maladaptive effects [18, 19]. In addition, autophagy has been identified as a crucial player in the inflammatory response and immunoreactivity associated with chronic respiratory conditions, including intermittent hypoxia and severe asthma [20, 21]. Notably, the overactivation of RAS has been implicated in triggering autophagy [22, 23]. Based on these observations, we postulate that Ang-(1-7) might regulate the CIH-exacerbated airway remodelling through the autophagy pathway. This study was aimed to explore how CIH worsens asthma and to elucidate the mechanism underlying the protective effects of Ang-(1-7) on airway remodelling, with a specific focus on autophagy.

## Results

### Ang-(1-7) decreased CIH-potentiated inflammation level, EMT and autophagy in OVA-challenged mice

**Fig. 1:**
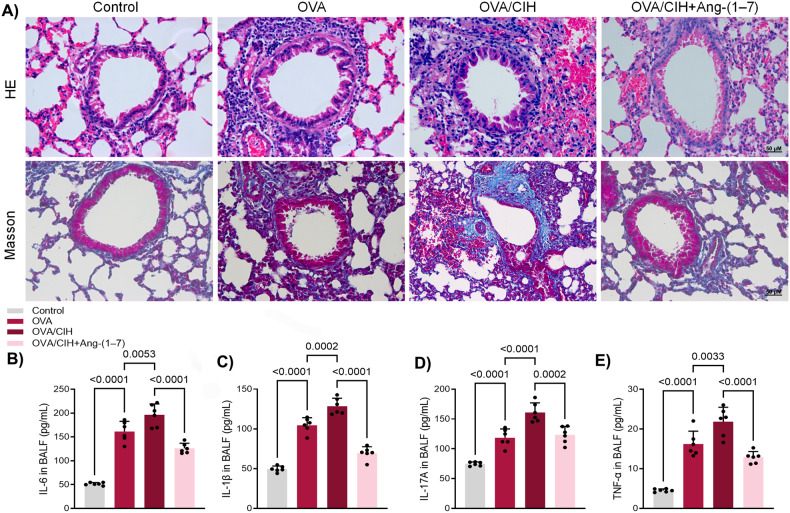
Ang-(1-7) suppressed CIH-enhanced lung injury and inflammation level in OVA- sensitised mice. **A** H&E staining and Masson’s trichrome staining results of mice with OVA or OVA + CIH or OVA + CIH + Ang-(1-7). **B** ELISA results of IL-6 in BALF. **C** ELISA results of IL-1β in BALF. **D** ELISA results of IL-17A in BALF. **E** ELISA results of TNF-α in BALF. Data are expressed as mean ± SD (*n* = 6).

Both haematoxylin and eosin (H&E) staining and Masson’s trichrome staining revealed that ovalbumin (OVA) induced airway injury in mice, CIH exposure intensified the level of injury while Ang-(1-7) administration significantly mitigated CIH’s impact on lung injury (Fig. 1A). In bronchoalveolar lavage fluid (BALF), inflammatory biomarkers including interleukin (IL)-6, IL-1β, IL-1, IL-17A, and tumour necrosis factor-α (TNF-α) were elevated in OVA-challenged mice, with even higher levels in the OVA + CIH group. Ang-(1-7) treatment effectively suppressed the overexpression of these biomarkers (Fig. 1B–E).

**Fig. 2:**
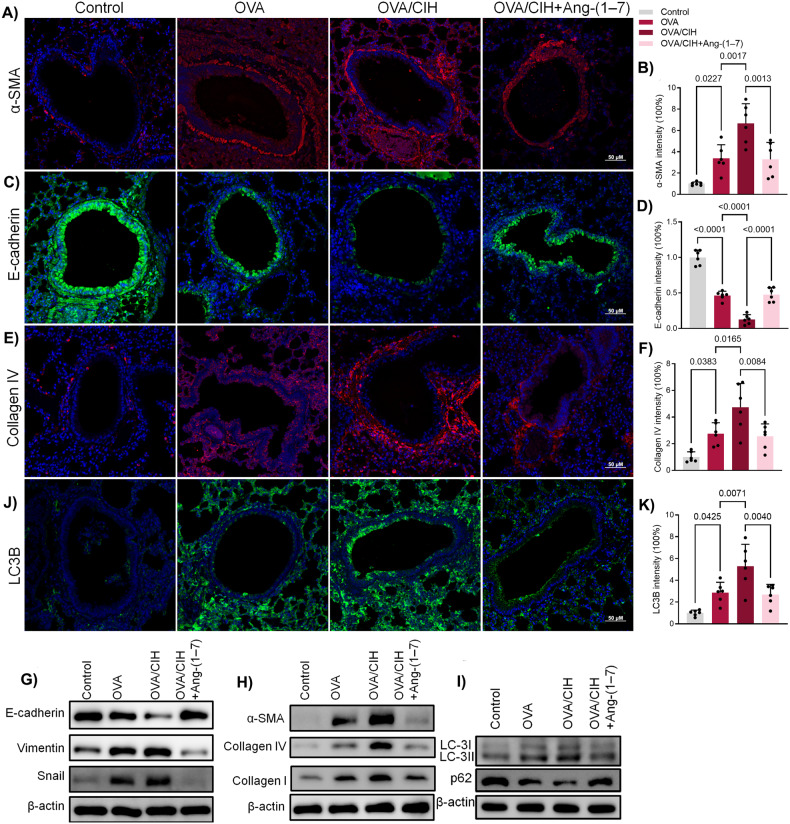
Ang-(1-7) decreased CIH-potentiated EMT and pulmonary fibrosis, and autophagy in OVA-challenged mice. **A** Immunofluorescence results of α-SMA in mice with OVA or OVA + CIH or OVA + CIH+Ang-(1-7). **B** Quantitative analysis of α-SMA. **C** Immunofluorescence results of E-cadherin. **D** Quantitative analysis of E-cadherin. **E** Immunofluorescence results of Collagen IV. **F** Quantitative analysis of Collagen IV. **G** Western blot results of E-cadherin, Vimentin and Snail. **H** Western blot results of α-SMA, Collagen IV and Collagen I. **I** Western blot results of LC3B and p62. **J** Immunofluorescence results of LC3B in mice with OVA or OVA + CIH or OVA + CIH + Ang-(1-7). **K** Quantitative analysis of LC3B. Data are expressed as mean ± SD (*n* = 6).

Immunofluorescence results showed that OVA-induced asthma increased α-smooth muscle actin (α-SMA) and Collagen IV expression, while inhibiting the expression of E-cadherin (Fig. 2A–F). CIH treatment significantly intensified the inhibitory effect of OVA on E-cadherin, while simultaneously resulting in an intensified expression of α-SMA, Collagen IV, and Collagen I. Western blotting confirmed these findings, showcasing that CIH exposure, along with OVA, augmented EMT and fibrosis-related proteins beyond OVA alone. And Ang-(1-7) treatment successfully counteracted these effects (Fig. 2G, H and Fig. S1A, B).

LC3B, a central protein in the autophagy pathway where it functions in autophagy substrate selection and autophagosome biogenesis, was intensified by OVA and further boosted by CIH + OVA, but attenuated by Ang-(1-7) (Fig. 2J, K). Western blotting further confirmed that OVA directly led to overexpression of LC3B-I and LC3B-II while inhibiting p62 expression (Fig. 2I and Fig. S1C). CIH heightened these effects, and Ang-(1-7) effectively reversed these enhancements (Fig. 2I and Fig. S1C).

These results suggest that CIH enhanced OVA-induced effects on EMT, fibrosis, and autophagy, and all of these effects were effectively reversed by Ang-(1-7).

### Ang-(1-7) dose-dependently suppressed CIH-potentiated EMT and autophagy in LPS-induced human bronchial epithelial cells

**Fig. 3:**
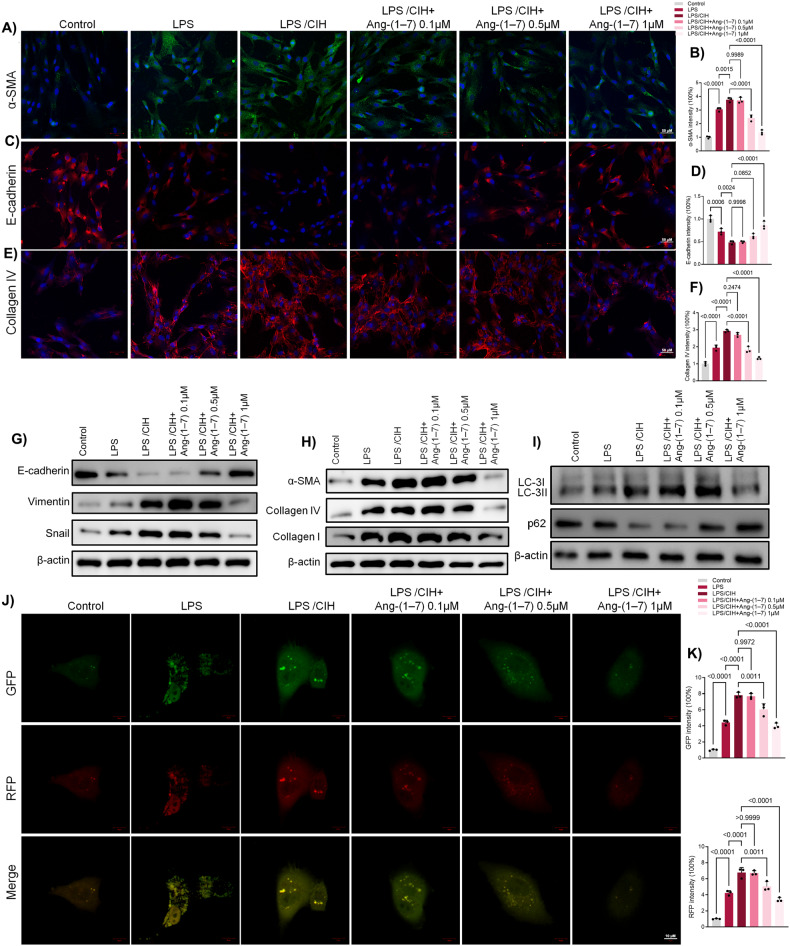
Ang-(1-7) dose-dependently suppressed CIH-potentiated EMT and pulmonary fibrosis in LPS-induced human bronchial epithelial cells by mediating autophagy. **A** Immunofluorescence results of α-SMA in human bronchial epithelial cells (BEAS-2b) with different dose of Ang-(1-7). **B** Quantitative analysis of α-SMA. **C** Immunofluorescence results of E-cadherin. **D** Quantitative analysis of E-cadherin. **E** Immunofluorescence results of Collagen IV. **F** Quantitative analysis of Collagen IV. **G** Western blot results of E-cadherin, Vimentin and Snail. **H** Western blot results of α-SMA, Collagen IV and Collagen I. **I** Western blot results of LC3B and p62. **J** Representative images of immunofluorescence staining of mRFP-GFP-LC3 in human bronchial epithelial cells. Representative profiles of autophagosomes (RFP + GFP + dots) and autolysosomes (RFP + GFP - dots). **K** Quantitative analysis of mRFP-GFP-LC3. Data are expressed as mean ± SD (*n* = 3).

Bronchial epithelial cells, BEAS-2B, were subjected to lipopolysaccharides (LPS) or a combination of LPS and CIH. Concurrently, three other groups were treated with different doses of Ang-(1-7) (0.1 μM, 0.5 μM, 1 μM) after CIH. Immunofluorescence analysis showed that LPS increased α-SMA and Collagen IV, while reducing E-cadherin expression (Fig. 3A–F). CIH intensified LPS’s effects. 0.1 μM of Ang-(1-7) had minimal effects on each group, but with increasing doses, Ang-(1-7) gradually reversed all effects induced by CIH (Fig. 3A–F). Western blot analyses showed similar results for α-SMA, Collagen IV, Collagen I, E-cadherin, Vimentin and Snail after the same procedures (Fig. 3G, H, Fig. S1D–E).

Immunofluorescence findings indicated increased autophagosomes and autolysosomes due to LPS stimulation, with CIH amplifying the effects of LPS (Fig. 3J, K). However, as the dosage of Ang-(1-7) increased from 0.1 μM to 1 μM, the levels of autophagosomes and autolysosomes progressively declined (Fig. 3J, K). Western blot analyses revealed similar results of LC3B and p62 protein levels. LPS along with CIH increased the expression of LC3B while controlling p62, and Ang-(1-7) dose-dependently counteracted the effects of LPS/CIH on those autophagy-related proteins (Fig. 3I and Fig. S1F).

Additionally, BEAS-2B cells were exposed to human recombinant IL-4/IL-13/transforming growth factor-beta 1 (TGF-β1) along with CIH, serving as an alternative asthmatic cell model. Concurrently, three other groups were treated with varying doses of Ang-(1-7) (0.1 μM, 0.5 μM, 1 μM) after CIH exposure. Western blot analyses demonstrated a substantial rise in α-SMA, Collagen I and Collagen IV following IL-4/IL-13/TGF-β1 treatment combined with CIH exposure. Notably, Ang-(1-7) successfully suppressed the effects (Fig. S1G, H). Immunofluorescence results yielded similar results of α-SMA (Fig. S1K). Furthermore. IL-4/IL-13/TGF-β1 along with CIH increased the expression of LC3B while controlled p62, Ang-(1-7) dose-dependently inhibited the effects of IL-4/IL-13/TGF-β1 + CIH on those autophagy-related proteins (Fig. S1I, J).

Collectively, these findings demonstrate that Ang-(1-7) effectively, in a dose-dependent manner, diminishes CIH-amplified EMT and autophagy in LPS-induced human bronchial epithelial cells.

To confirm the therapeutic effects of Ang-(1-7), BEAS-2B cells were treated with A779, an Ang-(1-7) antagonist, before LPS exposure. Immunofluorescence showed increased levels of α-SMA and Collagen IV and decreased levels of E-cadherin after LPS induction and CIH, yet Ang-(1-7) effectively countered these effects. A779 reversed Ang-(1-7)’s effects on α-SMA, Collagen IV, and E-caherin (Fig. S2A-F). Furthermore, Western blotting confirmed Ang-(1-7) effectively suppressed the LPS + CIH-induced promotion of Vimentin, Snail, α-SMA and Collagen IV while A779 treatment inhibited the suppression by Ang-(1-7) (Fig. S2G, H and Fig. S3A, B).

Furthermore, Ang-(1-7) effectively inhibited the LPS-induced and CIH-boosted autophagosome and autolysosome increase, but A779 erased this effect (Fig. S2J, K). Western blotting confirmed LC3B expression increase and p62 decrease with A779 treatment (Fig. S2I and Fig. S3C). These findings collectively demonstrate Ang-(1-7)’s success in controlling LPS + CIH-induced EMT, epithelial fibrosis, and autophagy.

### Ang-(1-7) inhibited EMT by suppressing autophagy in LPS-induced human bronchial epithelial cells

**Fig. 4:**
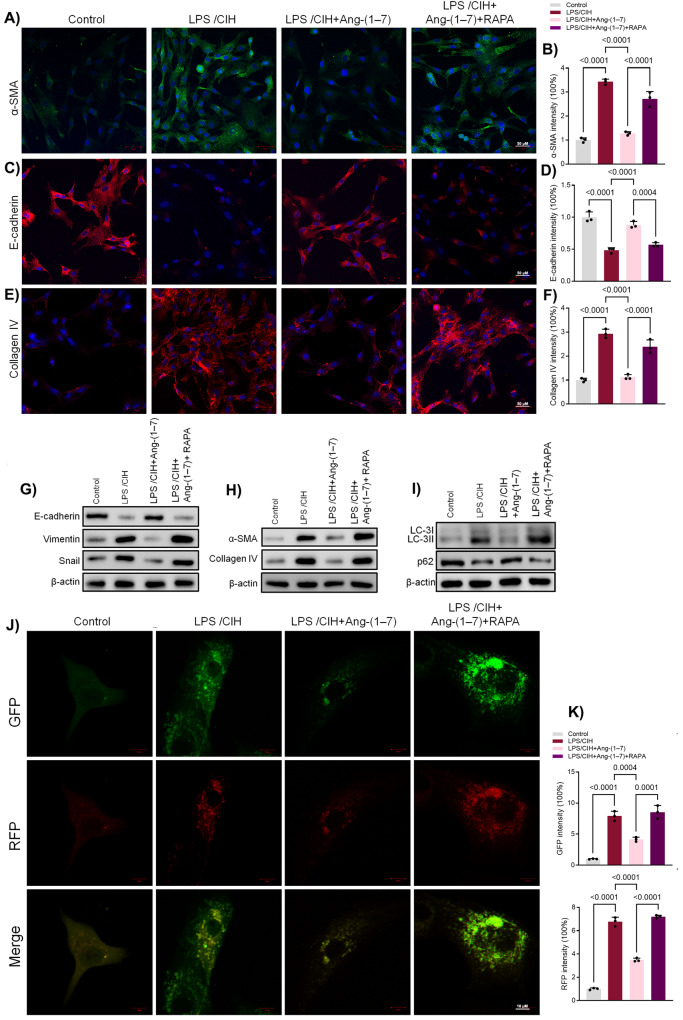
Rapamycin reversed the effects of Ang-(1-7) in LPS-induced human bronchial epithelial cells. **A** Immunofluorescence results of α-SMA in human bronchial epithelial cells with CIH, Ang-(1-7) or Ang-(1-7) along with RAPA. **B** Quantitative analysis of α-SMA. **C** Immunofluorescence results of E-cadherin. **D** Quantitative analysis of E-cadherin. **E** Immunofluorescence results of Collagen IV. **F** Quantitative analysis of Collagen IV. **G** Western blot results of E-cadherin, Vimentin and Snail. **H** Western blot results of α-SMA and Collagen IV. **I** Western blot results of LC3B and p62. **J** Representative images of immunofluorescence staining of mRFP-GFP-LC3 in human bronchial epithelial cells. Representative profiles of autophagosomes (RFP + GFP + dots) and autolysosomes (RFP + GFP − dots). **K** Quantitative analysis of mRFP-GFP-LC3. Data are expressed as mean ± SD (*n* = 3).

To further determine whether autophagy was associated with EMT, the cells were cultured with the autophagy-inducer rapamycin (RAPA). Immunofluorescence analysis revealed that RAPA significantly reversed Ang-(1-7)’s effects on α-SMA, Collagen IV and E-cadherin (Fig. 4A–F). In addition, Western blot results confirmed the promotion of Vimentin, Snail, α-SMA, and Collagen IV induced by LPS + CIH. Ang-(1-7) effectively suppressed the promotion, while RAPA treatment inhibited the suppression by Ang-(1-7) (Fig. 4G, H and Fig. S3D, E).

While Ang-(1-7) effectively inhibited the the increased levels of autophagosomes and autolysosomes induced by LPS and CIH, RAPA treatment nullified the suppressing effects of Ang-(1-7) and increased the levels (Fig. 4J, K). Western blotting confirmed increased LC3B expression and reduced p62 expression after RAPA treatment (Fig. 4I and Fig. S3F). Collectively, these findings suggest that RAPA attenuated the effects of Ang-(1-7) in LPS-induced human bronchial epithelial cells by enhancing autophagy.

### Ang-(1-7) inhibited autophagy via the HIF-1α/THBS1 axis in LPS-induced human bronchial epithelial cells

**Fig. 5:**
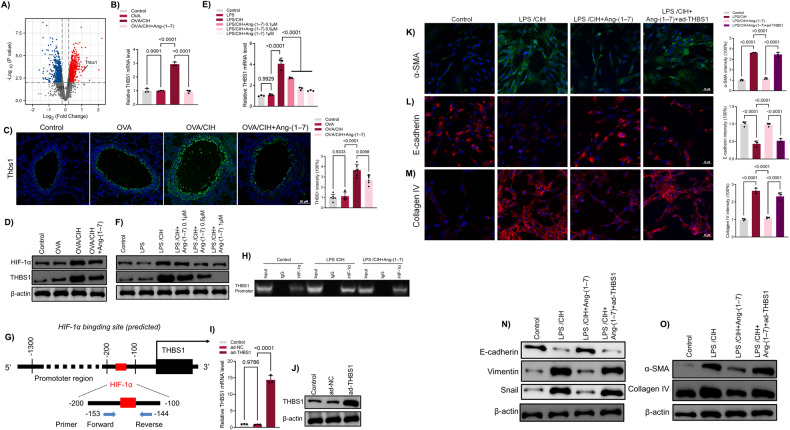
Ang-(1-7) inhibited EMT and fibrosis in LPS-induced human bronchial epithelial cells via HIF-1α/THBS1 axis. **A** Volcano plot of differential expressed genes. **B** qRT-PCR results of THBS1 in mice lung tissues. **C** Immunofluorescence results of THBS1 in mice with OVA or OVA + CIH or OVA + CIH + Ang-(1-7) and quantitative analysis of THBS1. **D** Western blot results of HIF-1α and THBS1 in mice lung tissues. **E** qRT-PCR results of THBS1 in human bronchial epithelial cells. **F** Western blot results of HIF-1α and THBS1 in human bronchial epithelial cells. **G** Jaspar predicts a putative HIF-1α binding site locating on the promoter region of THBS1. **H** ChIP assay of the relative enrichment of HIF-1α on the promoter region of THBS1. **I** qRT-PCR results of THBS1 in human bronchial epithelial cells infected with adenovirus overexpressing THBS1 (ad-THBS1). **J** Western blot results of THBS1 in human bronchial epithelial cells. **K** Immunofluorescence results of α-SMA in human bronchial epithelial cells with CIH, Ang-(1-7) or Ang-(1-7) infected with ad-THBS1. **L** Immunofluorescence results of E-cadherin and quantitative analysis of E-cadherin. **M** Immunofluorescence results of Collagen IV and quantitative analysis of Collagen IV. **N** Western blot results of E-cadherin, Vimentin and Snail. **O** Western blot results of α-SMA and Collagen IV. Data are expressed as mean ± SD (*n* = 3).

To explore how CIH contributes to EMT through autophagy in asthma, we analysed the different transcriptomic data between normal mouse lung tissues and those exposed to CIH (GEO accession no. GSE21409). The volcano plot indicates significant increases in hypoxia-inducible factor 1-alpha (HIF-1α) and Thrombospondin 1 (THBS1) levels after CIH exposure (Fig. 5A). The qRT-PCR, Western blot analyses, and immunofluorescence results confirmed THBS1 elevation in the OVA + CIH group, effectively suppressed by Ang-(1-7) treatment (Fig. 5B–D and Fig. S3G). Similarly, Ang-(1-7) suppressed CIH-elevated THBS1 level in LPS-induced human bronchial epithelial cells (Fig. 5E, F and Fig. S3H). Notably, HIF-1α functions as a transcription factor. Leveraging the JASPAR database (https://jaspar.genereg.net/), we pinpointed a potential HIF-1α binding site on the THBS1 promoter region (Fig. 5G). A chromatin immunoprecipitation (ChIP) assay demonstrated substantial HIF-1α enrichment on the THBS1 promoter in CIH-exposed BEAS-2B cells (Fig. 5H). Intriguingly, Ang-(1-7) treatment notably diminished HIF-1α binding to the THBS1 promoter.

**Fig. 6:**
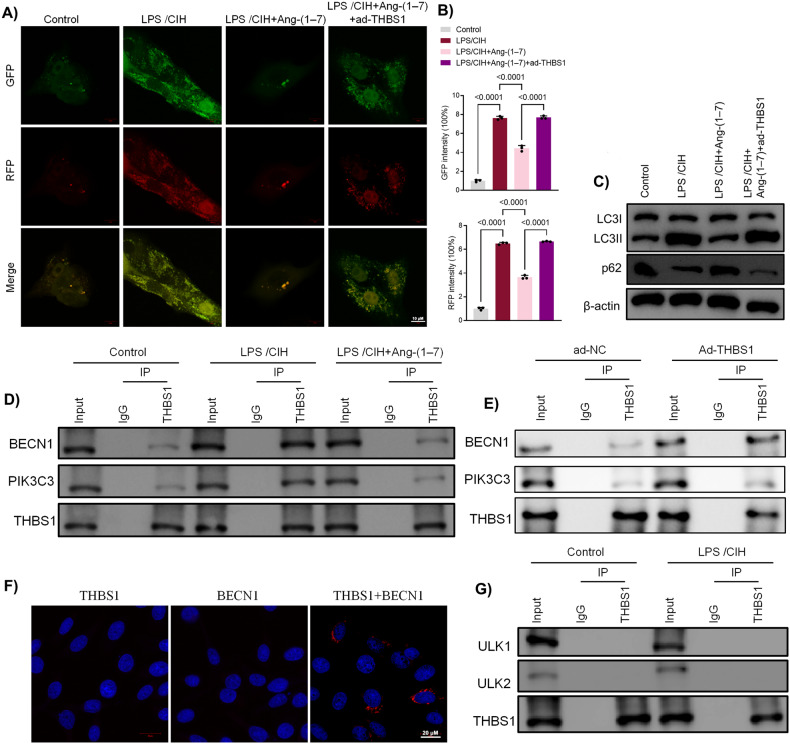
Ang-(1-7) inhibited autophagy in LPS-induced human bronchial epithelial cells via HIF-1α/THBS1 axis. **A** Representative images of immunofluorescence staining of mRFP-GFP-LC3 in human bronchial epithelial cells. Representative profiles of autophagosomes (RFP + GFP + dots) and autolysosomes (RFP + GFP − dots). **B** Quantitative analysis of mRFP-GFP-LC3. **C** Western blot results of LC3B and p62. **D** Interaction between THBS1 and BECN1-PIK3C3 detected by immunoprecipitation (IP) analysis in human bronchial epithelial cells with LPS/CIH or Ang-(1-7). **E** Interaction between THBS1 and BECN1-PIK3C3 detected by IP analysis in human bronchial epithelial cells. **F** Duolink PLA in situ assays in human bronchial epithelial cells. **G** Interaction between THBS1 and ULK1/ULK2 detected by IP analysis in human bronchial epithelial cells. Data are expressed as mean ± SD (*n* = 3).

Then we introduced adenovirus overexpressing THBS1 (ad-THBS1) to BEAS-2B cells. This resulted in a marked elevation in THBS1 mRNA and protein levels (Fig. 5I, J). Immunofluorescence results showed that ad-THBS1 significantly reversed the inhibiting effects of Ang-(1-7) on α-SMA and Collagen IV which were overexpressed after LPS/CIH (Fig. 5K, M). As for immunofluorescence results of EMT-related protein E-cadherin, the results showed ad-THBS1 attenuated the effects of Ang-(1-7) (Fig. 5L). Western blot results confirmed ad-THBS1 treatment reduced the inhibitory effects of Ang-(1-7) on EMT. (Fig. 5N, O and Fig. S3I, J). Besides, both immunofluorescence and Western blotting revealed that ad-THBS1 erased the suppressing effects of Ang-(1-7) on autophagy activation (Fig. 6A, B).

We next asked how THBS1 regulates autophagy during CIH exposure. Our findings revealed that LPS + CIH-induced promotion on the interaction between THBS1 and autophagy-associated protein Beclin 1 (BECN1), while Ang-(1-7) successfully suppressed this interaction. To validate the results, we examined whether the phosphatidylinositol 3-kinase (PtdIns3K) class III catalytic subunit (PIK3C3/Vps34), an important component of the BECN1 core complex, interacted with THBS1. As expected, the interaction between THBS1 and BECN1-PIK3C3 was evident in the context of LPS + CIH treatment (Fig. 6D). Furthermore, THBS1 overexpression strengthened the interaction between THBS1 and BECN1-PIK3C3 (Fig. 6E). Protein interactions were further validated by Duolink PLA labelled in red (Fig. 6F). Conversely, no interaction was detected between THBS1 and unc-51 like kinase 1 (ULK1) or ULK2, two pivotal proteins implicated in autophagy regulation, under control or LPS + CIH treatment condition (Fig. 6G).

### Exogenous THBS1 reversed the effect of Ang-(1-7) on suppressing CIH-enhanced lung injury in OVA- sensitised mice

**Fig. 7:**
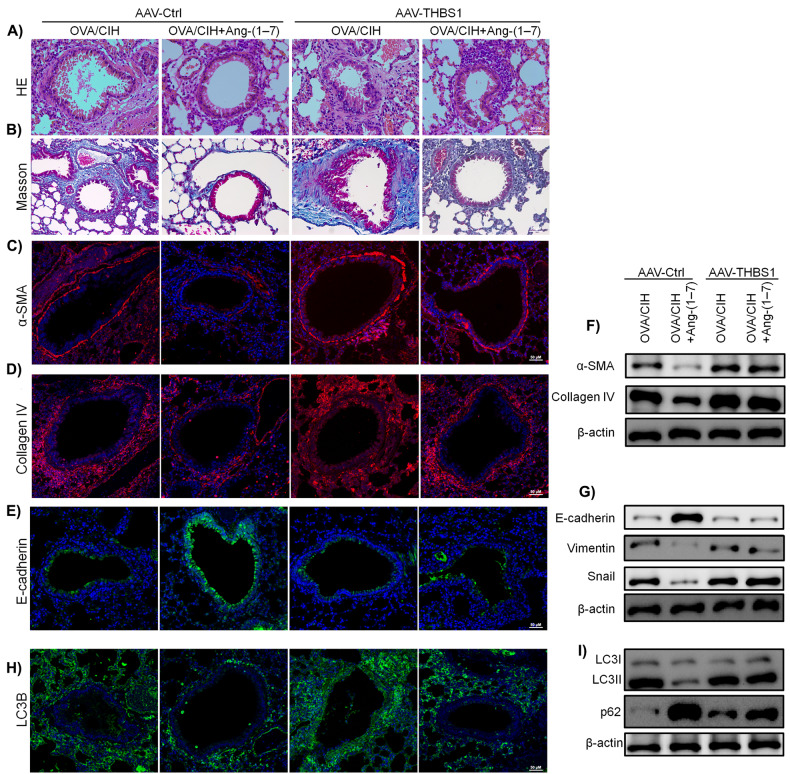
Exogenous THBS1 reversed the effect of Ang-(1-7) on suppressing CIH-enhanced lung injury in OVA- sensitised mice. **A**, **B** H&E staining and Masson’s trichrome staining results of mice injected with adeno-associated viruses of Control (AAV-Ctrl) and THBS1 (AAV-THBS1). **C** Immunofluorescence results of α-SMA in mice injected with AAV-Ctrl or AAV-THBS1. **D** Immunofluorescence results of Collagen IV. **E** Immunofluorescence results of E-cadherin. **F** Western blot results of α-SMA and Collagen IV. **G** Western blot results of E-cadherin, Vimentin and Snail. **H** Immunofluorescence results of LC3B. **I** Western blot results of LC3B and p62. Data are expressed as mean ± SD (*n* = 6).

To validate the mechanism of THBS1 in vivo, we evaluated OVA + CIH-induced asthma in THBS1-overexpressed mice by injecting adeno-associated viruses (AAV-THBS1). Compared with control group (AAV-Ctrl), THBS1-overexpressed mice significantly reversed the lung injury amelioration mediated by Ang-(1-7) (Fig. 7A, B). Immunofluorescence findings for α-SMA, Collagen IV and E-cadherin showed that AAV-THBS1 significantly reversed the inhibiting effects of Ang-(1-7) on lung EMT and fibrosis (Fig. 7C–E). Furthermore, Western blot results confirmed Ang-(1-7) effectively suppressed expression of EMT markers while AAV-THBS1 treatment inhibited the suppression by Ang-(1-7) (Fig. 7F, G and Fig. S3L, M). Next, immunofluorescence results confirmed that AAV-THBS1 nullified the inhibitory effects of Ang-(1-7) on LC3B level (Fig. 7H). Western blot results further demonstrated that LC3B expression increased and p62 expression decreased upon AAV-THBS1 administration (Fig. 7I and Fig. S3N)

## Discussion

This is the first study which elucidated how CIH, the main pathophysiological characteristics of OSA, aggravates airway remodelling through excess activation of autophagy. This study showed that, both murine and cellular asthma models, CIH-potentiated autophagy mediates EMT through HIF-1α/THBS1/BECN1 pathway, a process effectively countered by Ang-(1-7).

Strong and positive relationship between OSA and severe asthma has been revealed recently [24]. Airway remodelling is crucial in the development of severe asthma [25]. EMT and epithelial fibrosis are both defining features of airway remodelling. In parallel, Ang-(1-7) have emerged as a protective factor in diverse fibrotic conditions, including cardiac fibrosis, hepatic fibrosis, pulmonary fibrosis, and orthotopic breast tumours [26–29]. Furthermore, investigations have expounded upon Ang-(1-7)‘s inhibitory impact on EMT across liver, kidney, and lung contexts [13, 14, 30].

This study confirmed that CIH exposure increased OVA-induced pulmonary injury and inflammation levels. There was upregulation of EMT-related and fibrosis-related proteins, which could be reversed by Ang-(1-7) treatment. In vitro, Ang-(1-7) can dose-dependently inhibit CIH-enhanced in LPS (or Th2 cytokines and growth factors)-exposed human bronchial epithelial cells.

Cellular autophagy is essential for maintaining homoeostasis. The role of autophagy in the initiation and progression of asthma remains less understood. However, mounting evidence suggests that autophagy intricately regulates airway remodelling and EMT [31, 32]. The specific involvement of autophagy in CIH-potentiated airway remodelling has yet to be fully elucidated.

In this study, we found CIH triggered increased autophagy both in vivo and in vitro, evidenced by heightened LC3B levels and diminished p62 levels, and these effects were mitigated by ANG-(1-7). To further explore the role of autophagy in CIH-enhanced EMT, autophagy-inducer RAPA was used in the experiments. We observed that RAPA could reverse the effects of Ang-(1-7) on the expression of EMT and fibrosis markers. Likewise, the addition of A779, an Ang-(1-7) antagonist, resulted in outcomes similar to those of RAPA, confirming that Ang-(1-7) could attenuate CIH-aggravated EMT in both cellular and murine models of asthma by inhibiting autophagy.

We next investigated how CIH increased EMT through autophagy in asthma. Previous studies have indicated that Ang-(1-7) inhibits the expression of HIF-1α [33, 34]. This prompted us to investigate into HIF-1α and its potential downstream target genes. Analysis of GEO data revealed elevated HIF-1α and THBS1 expression in the lungs of CIH-exposed mice compared to those of normal mice. We further confirmed that the mRNA and protein levels of THBS1 and HIF-1α were elevated in CIH + OVA mice lung tissues as well as CIH + LPS-induced human lung bronchial epithelial cells. We further identified the binding site by using the JASPAR database [35], which was then verified by ChIP assay showing enrichment of HIF-1α on the THBS1 promoter region in CIH + LPS-induced human lung bronchial epithelial cells. Ang-(1-7) treatment markedly reduced the binding level. Further investigation via co-immunoprecipitation demonstrated that the interaction between THBS1 and the BECN1-PIK3C3 complex was enhanced upon CIH + LPS stimulation, and this interaction was impeded by Ang-(1-7) intervention. This indicates that Ang-(1-7) curtails CIH-induced autophagy by disrupting the interaction between THBS1 and the autophagy-associated protein BECN1.

THBS1, an adhesive glycoprotein, mediates cell-to-cell and cell-to-matrix interactions. Its involvement in tissue reconstruction, embryogenesis, angiogenesis, and tumour development has been documented [36, 37]. THBS1 possesses multiple binding sites that facilitate its interaction with a wide variety of other proteins [38]. Some studies suggest that THBS1 exerts an inhibitory effect on angiogenesis by binding to CD36 or CD47 [39, 40]. On the other hand, THBS1 plays a role in certain pathways that promote tumour development. For example, it is considered a major physiological activator of TGF-β1, and THBS1 promotes the EMT process in various cell types, including melanoma and oral squamous cell carcinoma, by activating TGF-β1 [41, 42]. Notably, recent studies have highlighted THBS1’s role in regulating autophagy through PERK-ATF4, which induces cardiac atrophy [43]. Our findings confirm that THBS1 participates in CIH-potentiated autophagy by interacting with BECN1 in cellular and murine models of asthma.

In summary, this study demonstrates that CIH aggravates airway remodelling, and Ang-(1-7) mitigates the exacerbation of EMT and autophagy by targeting HIF-1α/THBS1 axis. The results reveal a novel mechanism underlying OSA-potentiated asthma and suggest a potential therapeutic target.

## Materials and methods

### Animals

Eight-week-old male C57BL/6 mice weighing 20 ~ 22 g were used. They were kept under specific pathogen-free conditions and fed on an OVA-free diet. This study strictly adhered to the guidelines outlined in the eighth edition of ‘The Guide for the Care and Use of Laboratory Animals’ published by The National Academies Press at 2101 Constitution Ave. NW, Washington, DC 20055, USA. All animal experimentation was conducted in Ruijin hospital, Shanghai Jiao Tong University School of Medicine. All surgeries were performed under sodium pentobarbital anaesthesia, with all possible measures taken to reduce discomfort or suffering in the mice.

### Mice model establishment

All mice were randomised into the respective group. To establish the asthma model, mice were subjected to OVA-challenge alone or OVA-challenge along with CIH exposure. Mice in the control group were challenged with aerosolized saline and exposed to air. Specifically, mice were intraperitoneally sensitised with 0.2 mg of OVA (Grade V, Sigma, St Louis, MO, USA) complexed with 10 mg of alum in a total volume of 1 mL saline on day 0 and day 7. Control mice, on the other hand, were injected with alum in 1 mL saline. Starting from day 14, CIH or normal air exposures were initiated. Oxygen content in the CIH exposure chamber was monitored over several cycles using an oxygen sensor placed at the bottom of the chamber. During each 60-s cycle, animals were exposed to 5 s of 14 to 15% O2. These challenges were repeated every day for 8 h. Additionally, Ang-(1-7) (150 µg/kg) was administered to OVA-challenged mice exposed to CIH on Days 14, 21, 28, 35, and 42.

For in vivo overexpression of THBS1, custom-made adeno-associated viral vector carrying full-length cDNA of mice THBS1 (AAV6-THBS1) and its negative control (AAV9-Ctrl), were obtained from Hanbo Biotechnology Co. Ltd. (Shanghai, China). The adeno-associated virus was administered to mice via intratracheal injection at a concentration of 1 × 10^12^ vg/ml to achieve THBS1 overexpression. This injection was performed 7 days before OVA-challenge and repeated once a week. The modelling method was consistent with the procedure described above.

### Bronchoalveolar lavage fluid (BALF)

Twenty-four hours after the final CIH exposure, mice were humanely sacrificed by an overdose administration of pentobarbital (100 mg/kg intraperitoneal). The lungs were then lavaged three times via the endotracheal tube with 5 mL of phosphate buffer saline, and the lavage fluid was gently aspirated and pooled. BALF was centrifuged for 10 min (1000 g at 4 °C), and the supernatant was collected for further analysis.

### Lung histology

For histological examinations, the left lung lobe from mice was fixed in a 10% neutral-buffered formalin solution, embedded into paraffin, and cut into 5 μm sections which were subsequently subjected to H&E and Masson trichrome staining.

### Immunofluorescence

The xylol-deparaffinized samples were sectioned into 4 µm slices and then rehydrated using a graded series of ethanol. Antigen retrieval was performed using a heat-induced epitope protocol at 95 °C for 40 min. To block endogenous peroxidases, the sections were incubated in methanol containing 0.3% hydrogen peroxide. Samples were blocked with protein serum using a Vectastain Elite ABC kit (Vector Laboratories, Inc., Burlingame, CA, USA), then incubated overnight at 4 °C with anti-α-SMA, anti-E-cadherin, and anti-Collagen IV antibody (CST, MA, USA) at a dilution of 1:1000. After washing with TBST, the sections were exposed to a secondary antibody for 20 min at room temperature, followed by treatment with a peroxidase-conjugated biotin-streptavidin complex (Dako, Glostrup, Denmark) for 20 min. Visualisation was achieved using 3,3'-diaminobenzidine, and subsequent counterstaining was performed with hematoxylin. For the negative control, nonimmune serum was utilised in place of the primary antibody.

### Enzyme-linked immunosorbent assay (ELISA) in BALF

The concentrations of inflammatory cytokines IL-6, IL-1β, TNF-α and IL-17A in BALF were measured with an ELISA kit according to manufacturer´s instructions (Nanjing Jiancheng Bioengineering Institute).

### Cell culture

Human bronchial epithelial cells, BEAS-2B, were sourced from the American Type Culture Collection (Rockville, MD, USA) and cultured in Dulbecco’s Modified Eagle’s Medium containing 10% foetal calf serum at 37 °C with 5% CO_2_ and 95% air. Prior to LPS (10 μg/ml) or IL-4 (20 ng/mL)/IL-13 (20 ng/mL)/TGF-β1 (20 ng/mL) treatment for 48 h, Ang-(1-7) (1 μM), A779 (0.1 μM), or RAPA (0.1 μM) was added in BEAS-2B culture medium 30 min in advance. For CIH exposure, cells were subjected to 5 s of 14 to 15% O_2_ during every 60 s cycle for 24 h. The full-length cDNA of mice THBS1 adenovirus was purchased from Hanbio Biotechnology Co., Ltd. (Shanghai, China) and employed to infect primary hepatocytes. Supernatant from HEK-293T cells was collected and utilised for BEAS-2B infection using polybrene at a concentration of 10 μg/mL. Infected BEAS-2B cells were treated with puromycin at a concentration of 2 μg/mL to establish stable cell lines.

### Protein extraction and western blotting

Proteins extracted from subconfluent cell cultures and lung tissues were subjected to Western blot analyses. The primary antibodies against α-SMA (#19245), Snail (#3879), E-cadherin (#14472), Collagen IV (#50273), Collagen I (#72026), LC3B (#3868), p62 (#23214), ULK1 (#8054), ULK2 (ab97695, purchased from Abcam), THBS1 (#37879), BECN1 (#4122), PIK3C3 (#4263) and β-actin (#3700) were purchased from Cell Signalling Technology (Danvers, MA, USA). Signals were detected using a FluorChem E system (Alpha Innotech Corp, Santa Clara, CA, USA).

### RNA extraction and quantitative real-time PCR (qRT-PCR)

Total RNA was extracted from BEAS-2B cells or mice lung tissues using Trizol reagent and reverse transcribed into cDNA with a PrimeScript RT Master Mix Perfect Real-Time kit. Quantitative real-time PCR was performed using FastStart Universal SYBR-Green Master (Rox) and carried out on an ABI 7500 real-time PCR system (Applied Biosystems, Foster, CA, USA) using the following primers. Human β-actin forward primer, 5'-GTCCCTCACCCTCCCAAAAG-3'; reverse primer, 5'-GCTGCCTCAACACCTCAACCC-3'. Human THBS1 forward primer, 5'-AGACTCCGCATCGCAAAGG-3'; reverse primer, 5'-TCACCACGTTGTTGTCAAGGG-3'; Mouse β-actin forward primer, 5'-ATATCGCTGCGCTGGTCGTC-3'; reverse primer, 5'-AGGATGGCGTGAGGGAGAGC-3'. Mouse THBS1 forward primer, 5'-CCTGCCAGGGAAGCAACAA-3'; reverse primer, 5'-ACAGTCTATGTAGAGTTGAGCCC-3'. β-actin was used as the reference gene. Relative quantitation of target genes was calculated by the 2 − ΔΔCt method as recommended by the manufacturer. Each experiment was repeated three times in three samples.

### Co-Immunoprecipitation (CO-IP)

BEAS-2B cells were transfected with the indicated plasmids for 24 h and lysed with IP buffer. After an ultrasonic bath and centrifugation (12,000 g for 5 min), supernatants were incubated with protein A/G agarose beads (Thermo Scientific, USA) and anti‐tag antibody overnight at 4 °C. Beads were washed with NaCl buffer and boiled with SDS loading buffer for 15 min at 95 °C before Western blotting.

### Chromatin immunoprecipitation (ChIP)

BEAS-2B Cells were washed twice with PBS and cross-linking was performed in 9 mL culture medium containing 1% formaldehyde at room temperature for 15 min. The reaction was halted by adding glycine to the final concentration of 125 mM. Chromatin extraction was performed with the High Sensitivity ChIP Kit (Abcam) as per the manufacturer’s instructions. A total of 2 μg chromatin was used for the ChIP with anti-HIF-1α antibody overnight at 4 °C. Following cross-link reversal and DNA purification, 1 μL of the eluted DNA was used for quantitative polymerase chain reaction with target region primers: 5'-GGATCGACCTGACTGAACCT-3' and 5'-CCTGGGATCCGAACGGATCT-3'.

### Proximity ligation assay (PLA)

BEAS-2B cells were fixed using 3.7% paraformaldehyde and then blocked with the blocking solution provided by the Duolink PLA kit (Olink Bioscience, Uppsala, Sweden) according to the manufacturer’s instructions. Briefly, the fixed cells were incubated with anti-THBS1 and anti-BECN1 antibody overnight. After multiple washings, cells were sequentially incubated with PLA probes, ligation solution and amplification solution at 37 °C. Subsequently, cover-slips were mounted.

### Autophagic flux analysis

BEAS-2B cells were transfected with mRFP‐GFP-LC3 for 24 h. After transfection, mRFP‐GFP‐LC3‐BEAS-2B cells were fixed with 4% paraformaldehyde and stained with 10 μmol/L Hoechst 33342. Cell images were obtained using an Operetta High Content Imaging System (Perkin‐Elmer) and analysed using Harmony analysis software (Perkin‐Elmer). Cells were detected using green fluorescent protein (GFP) or monomeric red fluorescent protein (mRFP). Puncta in autophagosome and autolysosomes were stained yellow and red, respectively, in merged images. The assessment of autophagic flux involved quantifying the increased percentage of red puncta in merged images.

### Statistical analysis

The results are presented as mean ± SD. Statistical significance was evaluated by one-way ANOVA and by Student’s *t*-tests. A *p-*value of less than 0.05 indicates statistical significance.

### Supplementary information

Supplementary figure legends

Figure S1

Figure S2

Figure S3

Uncroped blot

author-contribution-form

## Data Availability

Data available on request from the authors
