# Supplementary material for: Exogenous Ang-(1-7) inhibits autophagy via HIF-1α/THBS1/BECN1 axis to alleviate chronic intermittent hypoxia-enhanced airway remodelling of asthma
Source: Cell Death Discov. 2023 Oct 2;9:366. doi: 10.1038/s41420-023-01662-0 (PMC10545676; doi:10.1038/s41420-023-01662-0)
Supplement: Supplementary file 1 — Supplementary figure legends [file 41420_2023_1662_MOESM1_ESM.docx]

**FIGURE S1. Ang-(1-7) attenuated the effects of Ang-(1-7) in IL-4/IL-13/TGF-β1-induced human bronchial epithelial cells**

A) Quantification analysis of E-cadherin, Vimentin and Snail protein expression. B) Quantification analysis of α-SMA, Collagen I and Collagen IV protein expression. C) Quantification analysis of LC3B and p62. D) Quantification analysis of E-cadherin, Vimentin and Snail protein expression. E) Quantification analysis of α-SMA, Collagen I and Collagen IV protein expression. F) Quantification analysis of LC3B and p62. G) Western blot results of α-SMA, Collagen I and Collagen IV. H) Quantification analysis of α-SMA, Collagen I and Collagen IV protein expression. I) Western blot results of LC3B and p62. J) Quantification analysis of LC3B and p62. K) Immunofluorescence results of α-SMA in human bronchial epithelial cells cells with different dose of Ang-(1-7). L) Quantitative analysis of α-SMA. Data are expressed as mean ± SD (n=3).

**FIGURE S2. Ang-(1-7) antagonist A779 attenuated the effects of Ang-(1-7) in LPS-induced human bronchial epithelial cells**

A) Immunofluorescence results of α-SMA in human bronchial epithelial cells with CIH, Ang-(1-7) or Ang-(1-7) along with A779. B) Quantitative analysis of α-SMA. C) Immunofluorescence results of E-cadherin. D) Quantitative analysis of E-cadherin. E) Immunofluorescence results of Collagen IV. F) Quantitative analysis of Collagen IV. G) Western blot results of E-cadherin, Vimentin and Snail. H) Western blot results of α-SMA and Collagen IV. I) Western blot results of LC-3 and p62. J) Representative images of immunofluorescence staining of mRFP-GFP-LC3 in human bronchial epithelial cells. K) Quantitative analysis of mRFP-GFP-LC3. Data are expressed as mean ± SD (n=3). ***P < 0.001 compared to control.

**FIGURE S3. Quantification analysis of Western blot results**

A) Quantification analysis of E-cadherin, Vimentin and Snail protein expression. B) Quantification analysis of α-SMA and Collagen IV protein expression. C) Quantification analysis of LC3B and p62. D) Quantification analysis of E-cadherin, Vimentin and Snail protein expression. E) Quantification analysis of α-SMA and Collagen IV protein expression. F) Quantification analysis of LC3B and p62. G) Quantification analysis of HIF-1α and THBS1 protein expression. H) Quantification analysis of HIF-1α and THBS1 protein expression. I) Quantification analysis of E-cadherin, Vimentin and Snail protein expression. J) Quantification analysis of α-SMA and Collagen IV protein expression. K) Quantification analysis of LC3B and p62. L) Quantification analysis of E-cadherin, Vimentin and Snail protein expression. M) Quantification analysis of α-SMA and Collagen IV protein expression. N) Quantification analysis of LC3B and p62. Data are expressed as mean ± SD (n=3).
