# Supplementary figures and images for: Exogenous Ang-(1-7) inhibits autophagy via HIF-1α/THBS1/BECN1 axis to alleviate chronic intermittent hypoxia-enhanced airway remodelling of asthma
Source: Cell Death Discov. 2023 Oct 2;9:366. doi: 10.1038/s41420-023-01662-0 (PMC10545676; doi:10.1038/s41420-023-01662-0)

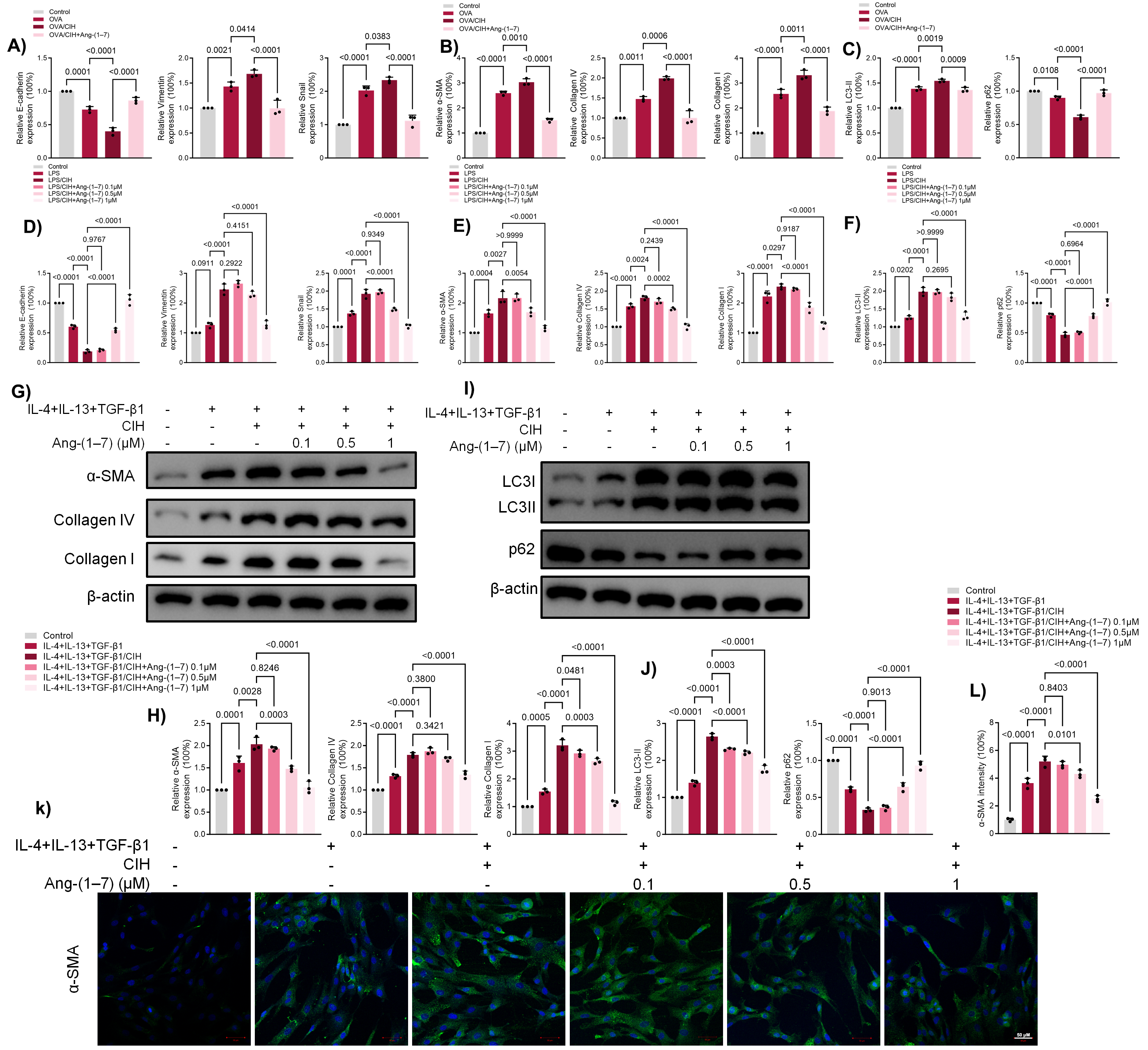

Supplement: Supplementary file 2 — Figure S1 [file 41420_2023_1662_MOESM2_ESM.tif]

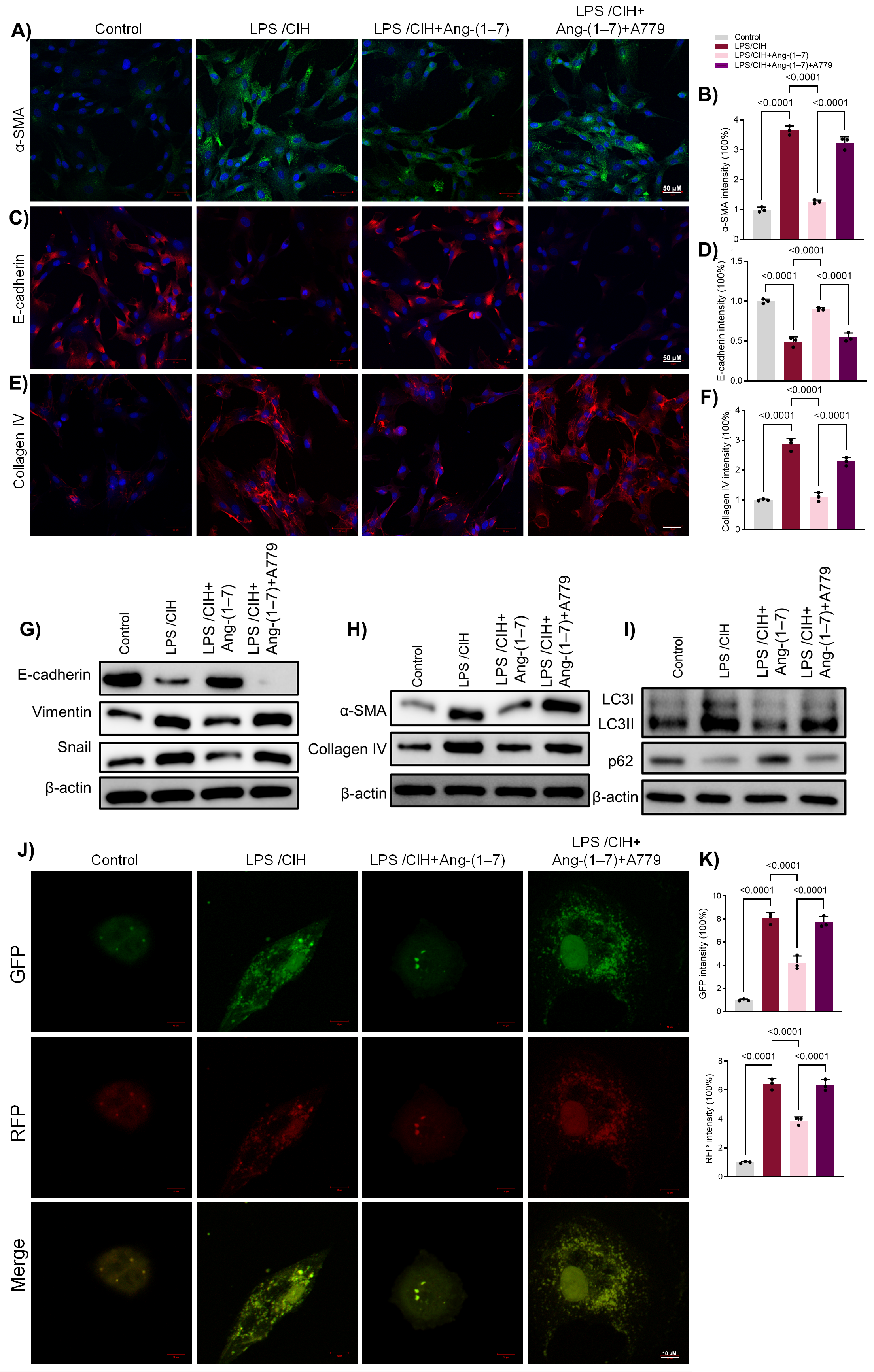

Supplement: Supplementary file 3 — Figure S2 [file 41420_2023_1662_MOESM3_ESM.tif]

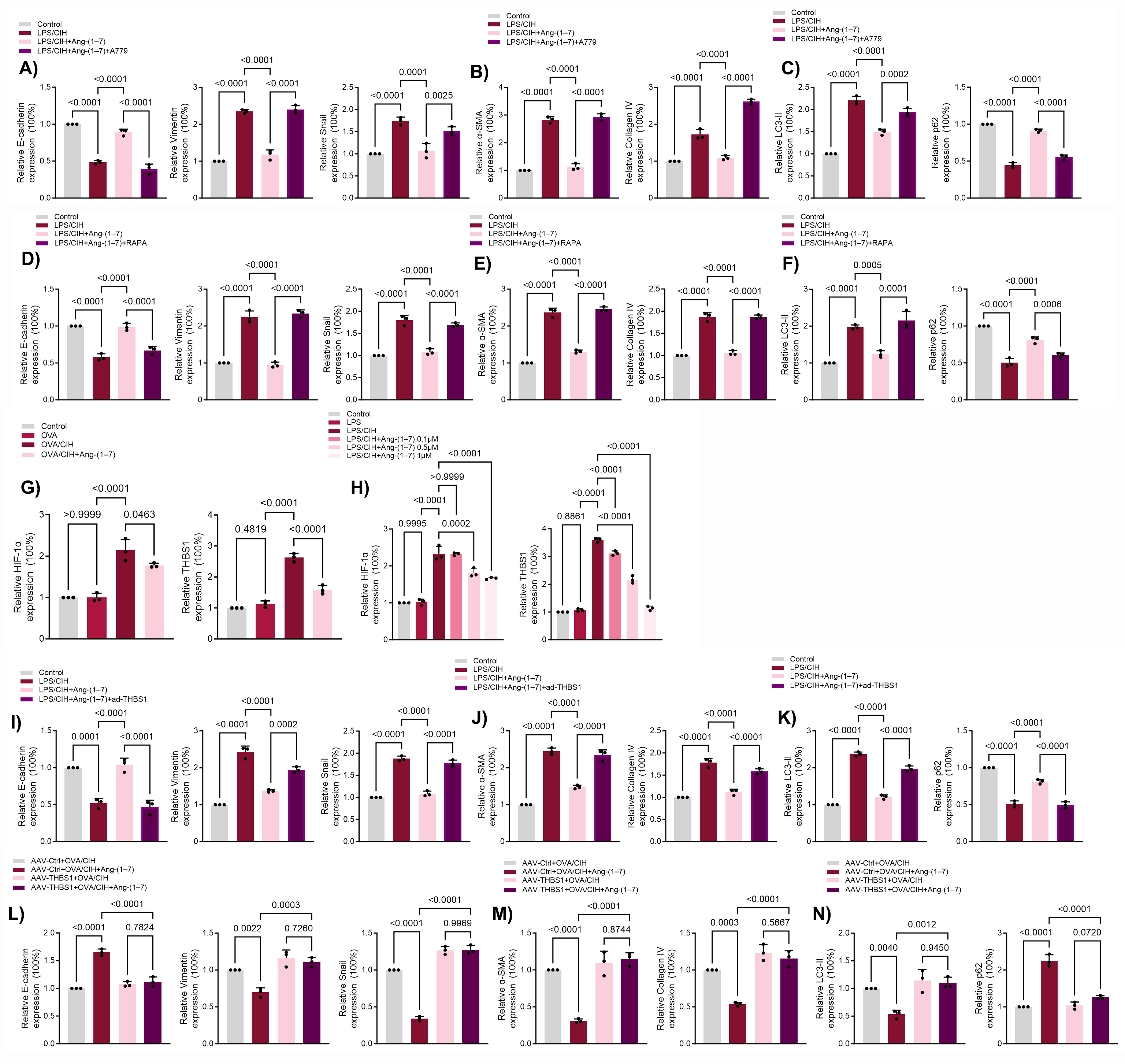

Supplement: Supplementary file 4 — Figure S3 [file 41420_2023_1662_MOESM4_ESM.tif]
