# Supplementary material for: Exogenous Ang-(1-7) inhibits autophagy via HIF-1α/THBS1/BECN1 axis to alleviate chronic intermittent hypoxia-enhanced airway remodelling of asthma
Source: Cell Death Discov. 2023 Oct 2;9:366. doi: 10.1038/s41420-023-01662-0 (PMC10545676; doi:10.1038/s41420-023-01662-0)
Supplement: Supplementary file 5 — Uncroped blot [file 41420_2023_1662_MOESM5_ESM.pdf]

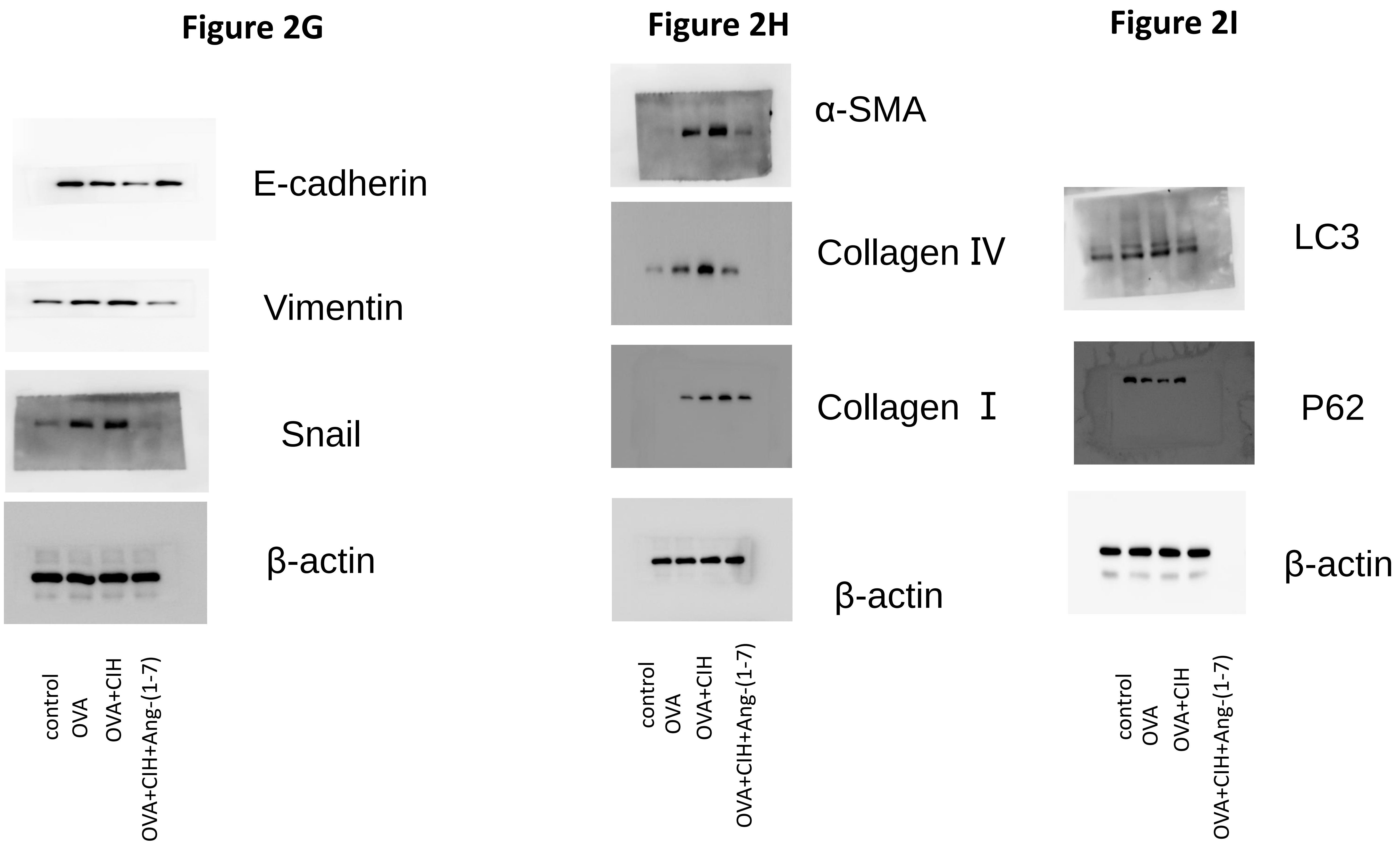
Figure 2G
E-cadherin
Vimentin
Snail
β-actin
control
OVA
OVA+CIH
OVA+CIH+Ang-(1-7)
Figure 2H
α-SMA
Collagen IV
Collagen Ⅰ
β-actin
control
OVA
OVA+CIH
OVA+CIH+Ang-(1-7)
Figure 2I
LC3
P62
β-actin
control
OVA
OVA+CIH
OVA+CIH+Ang-(1-7)

Figure 2

Figure 3G

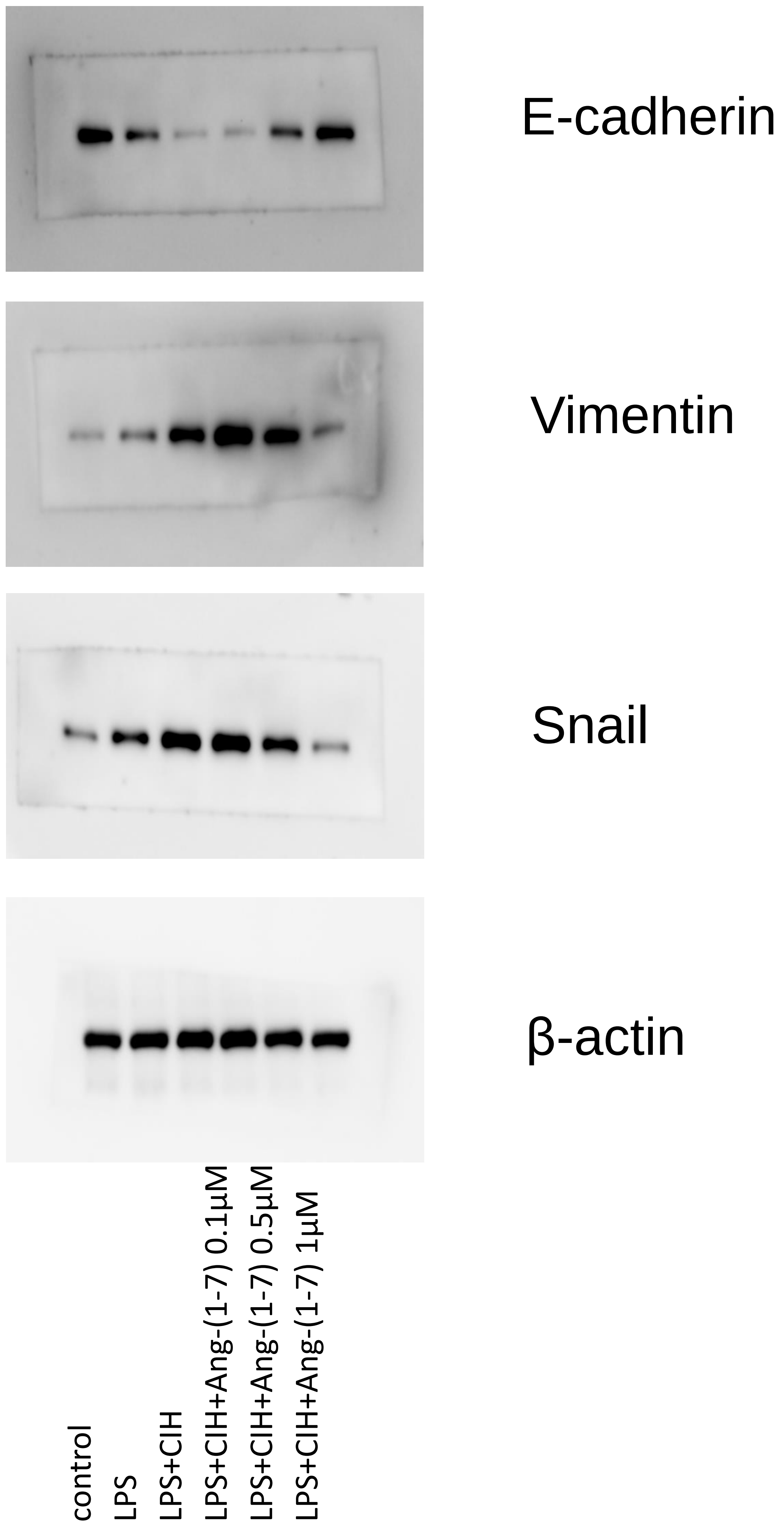

Figure 3H

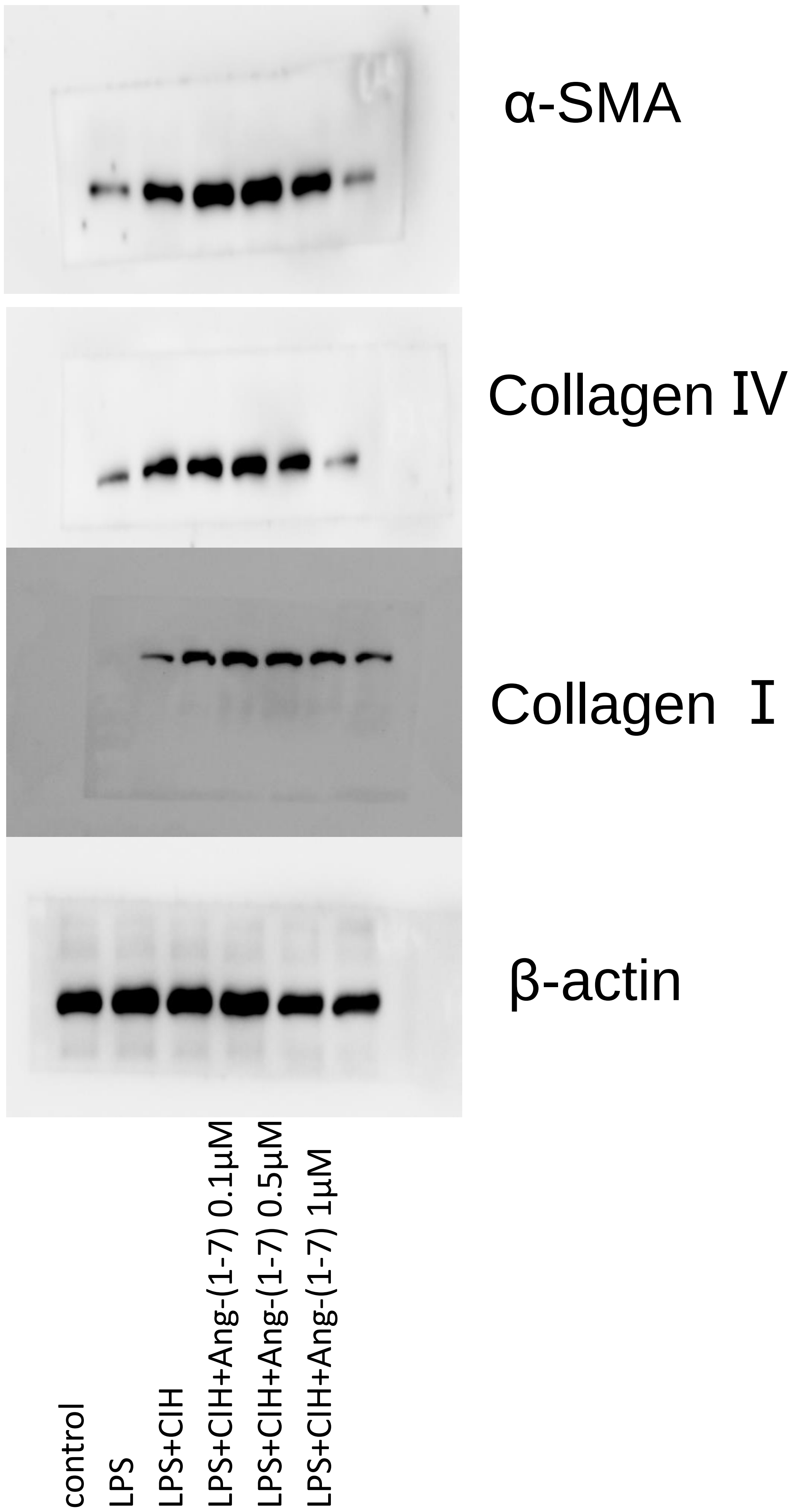

Figure 3I

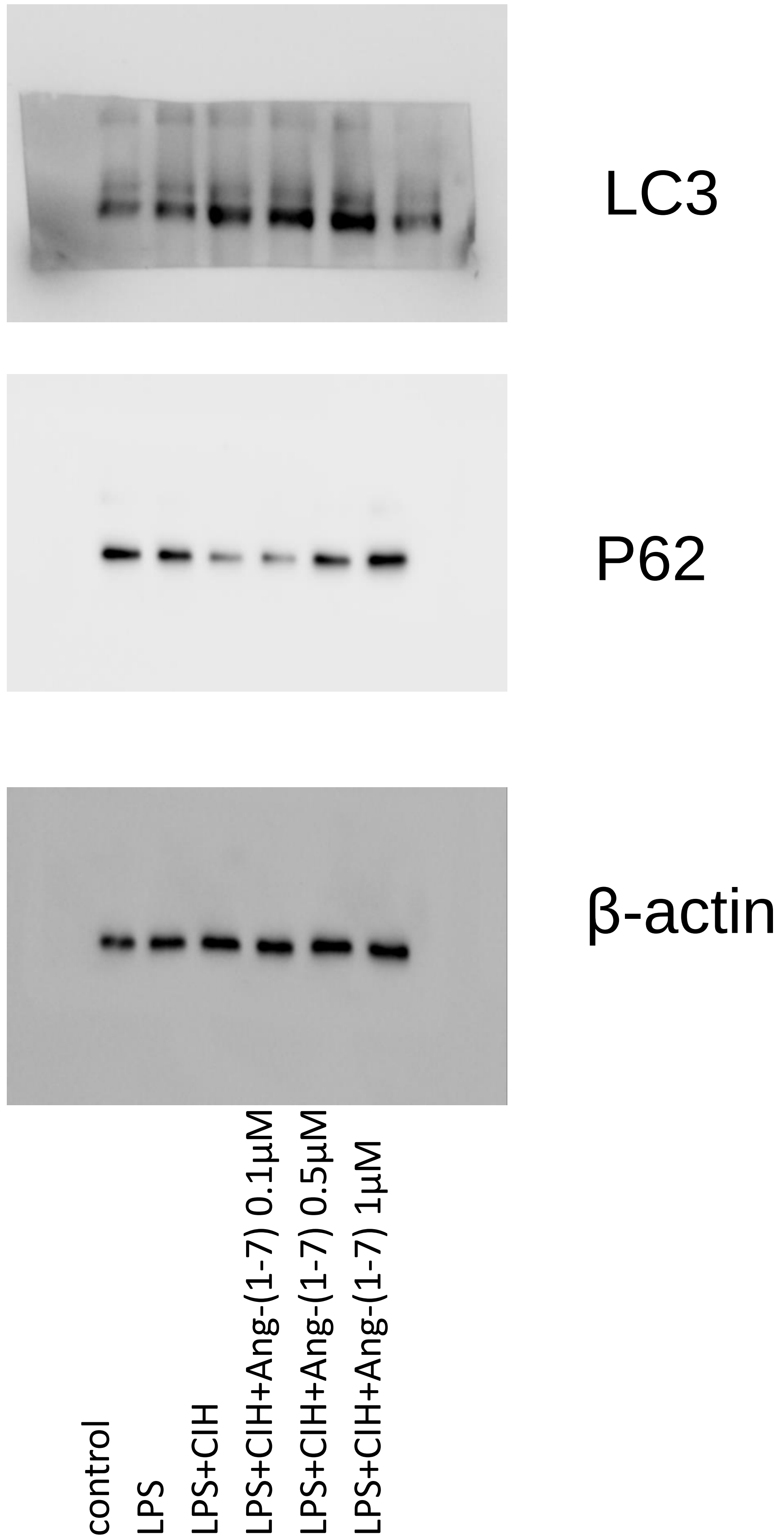

Figure 3

Figure 4G

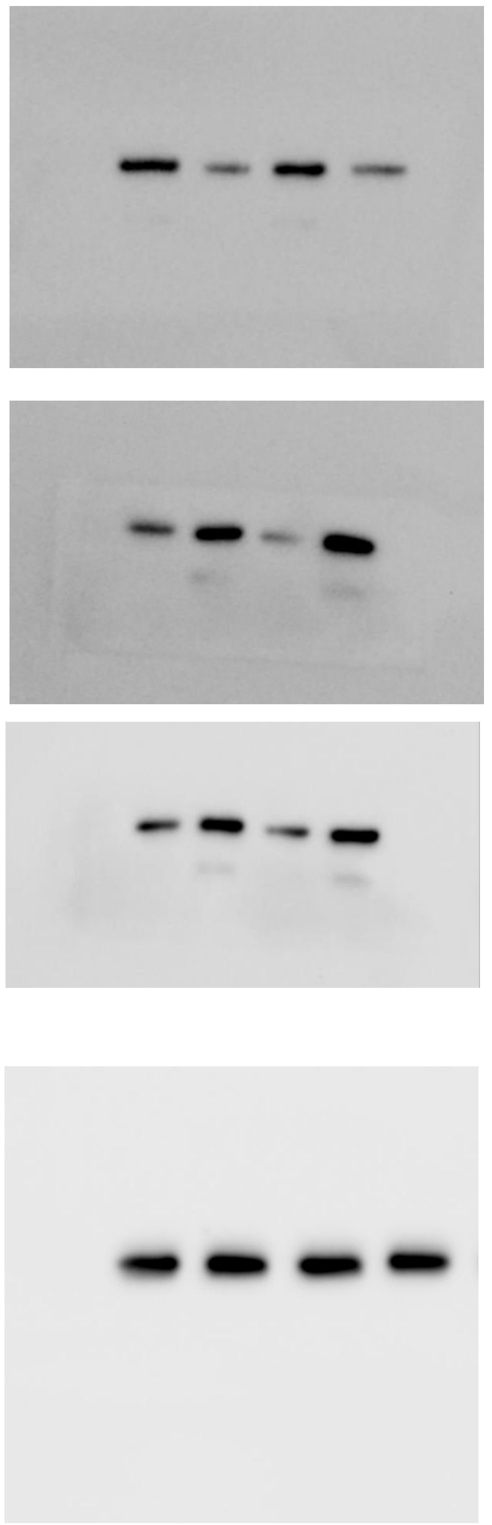

Figure 4H

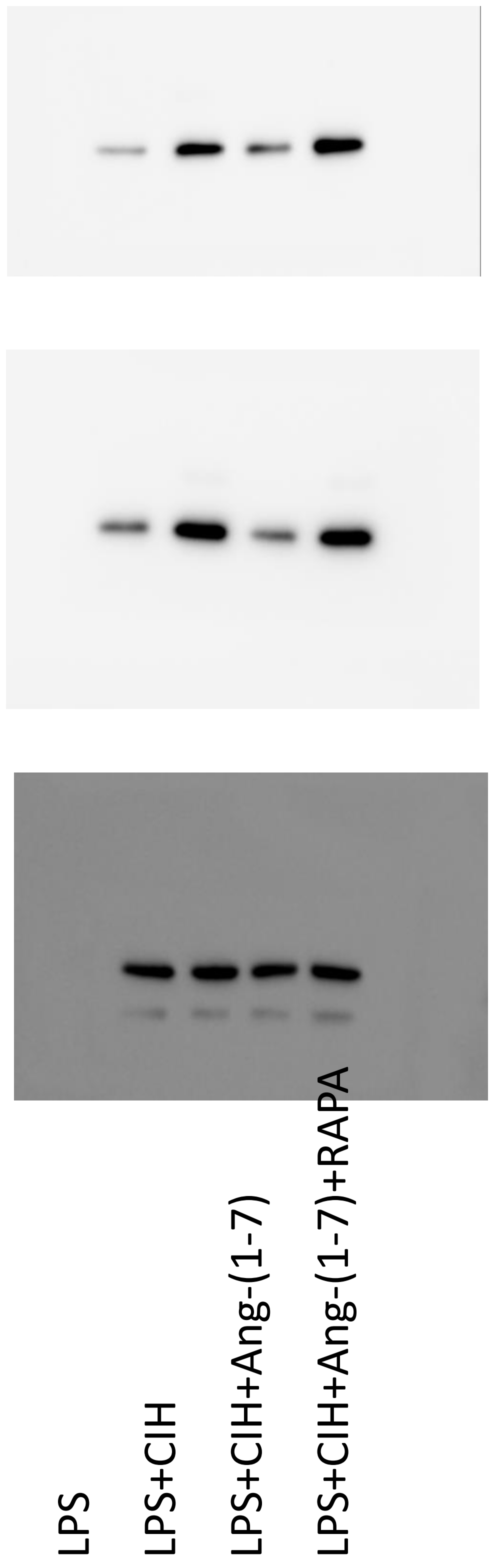

Figure 4I

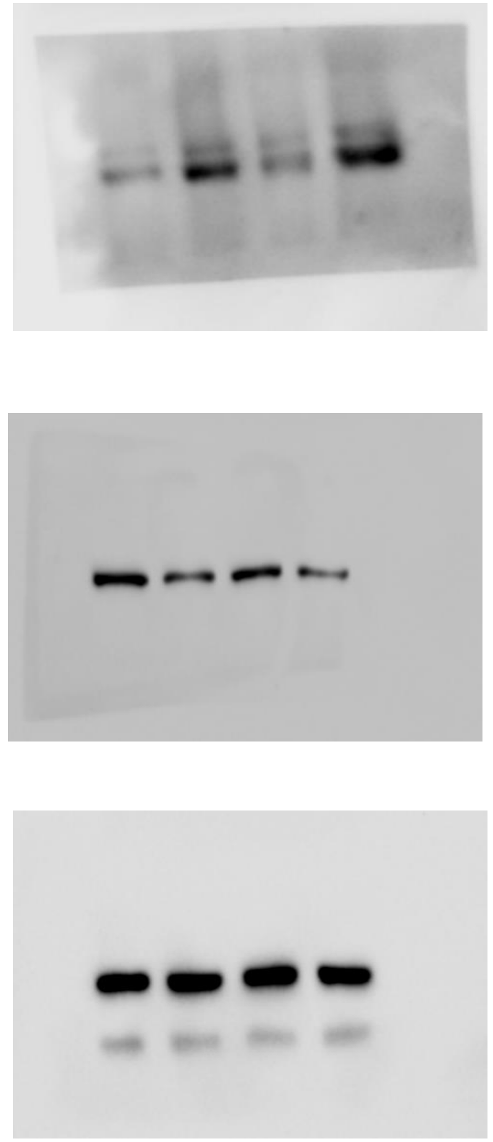

Figure 4

Figure 5F

Figure 5D

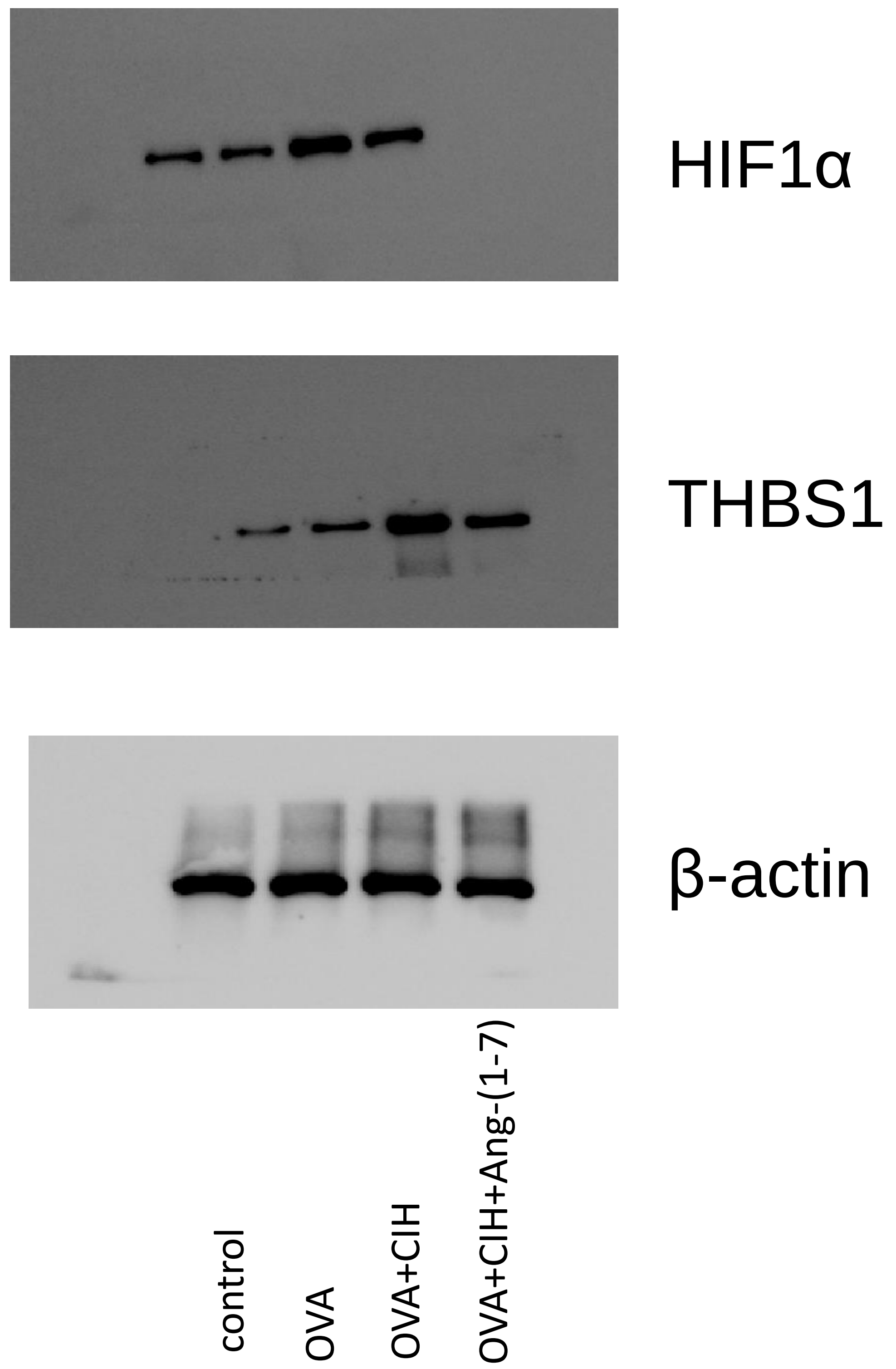

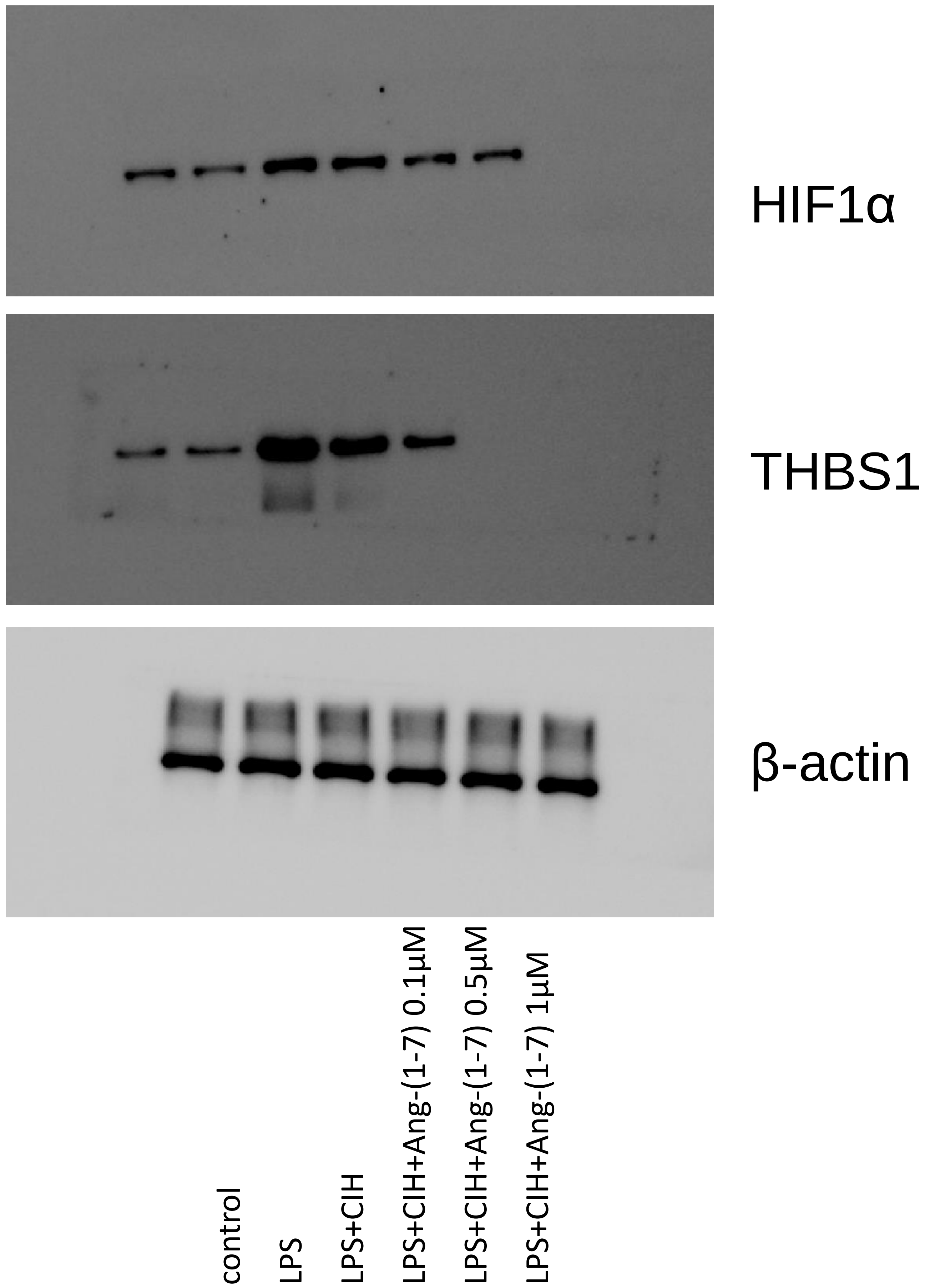

Figure 5J

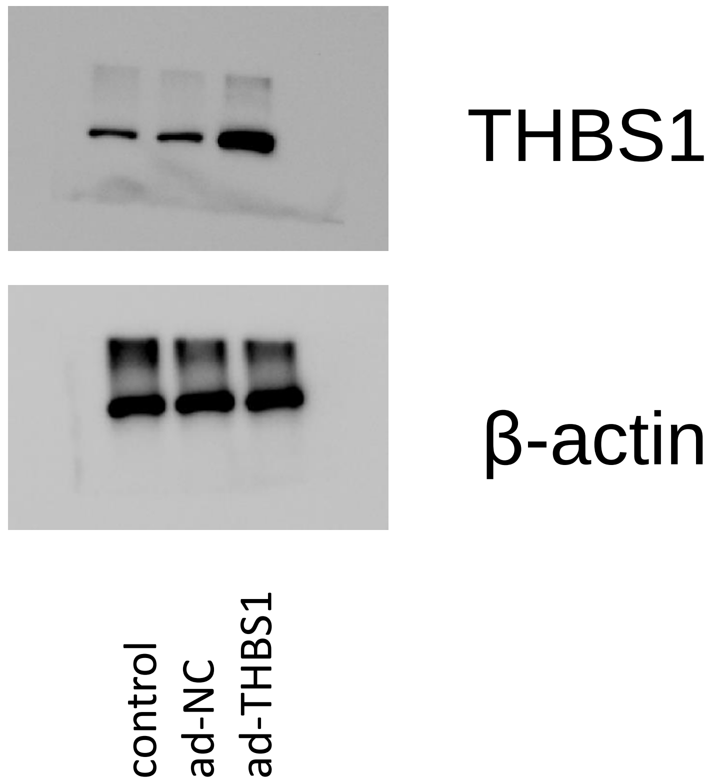

Figure 5

Figure 5N

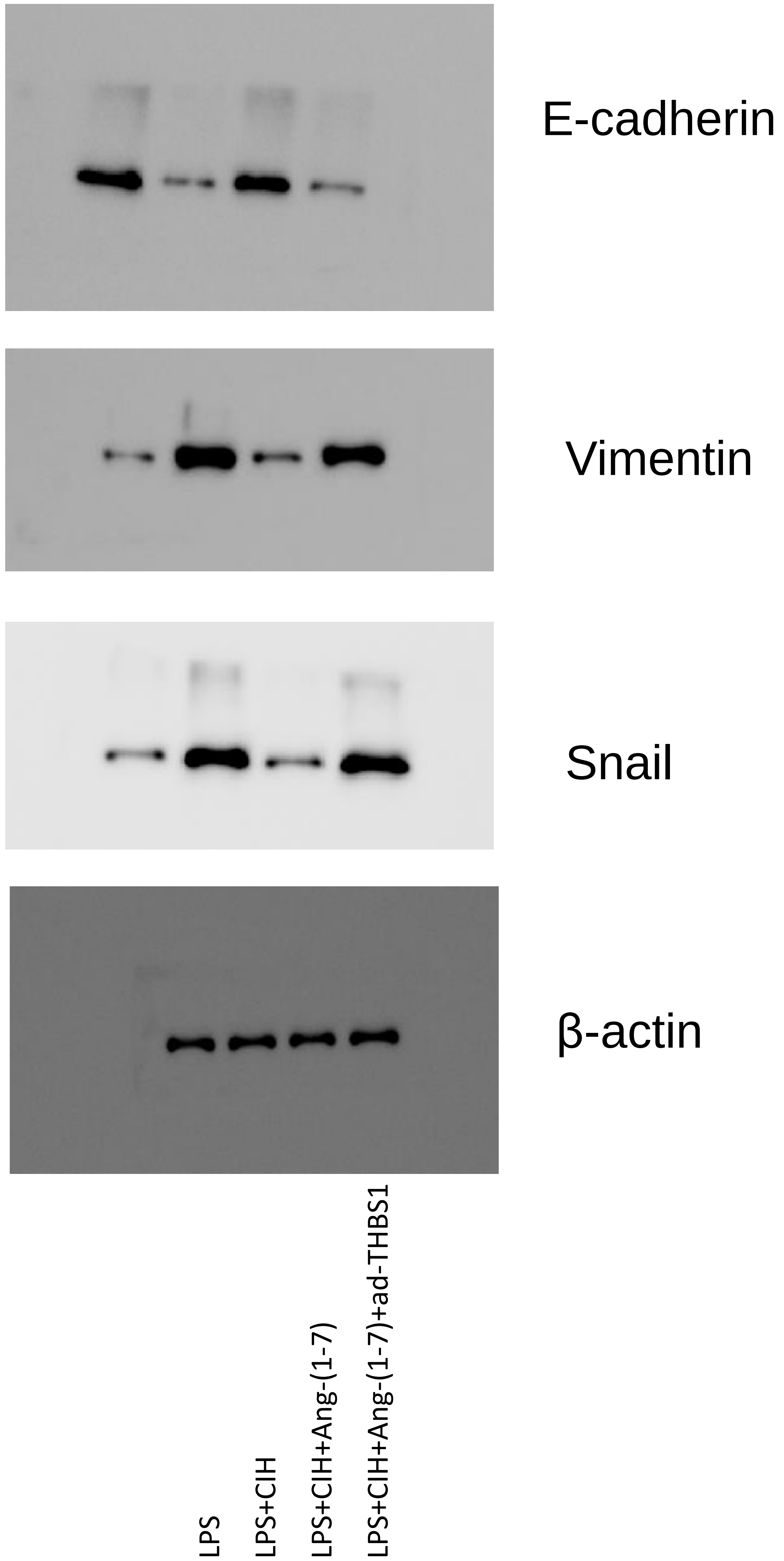

Figure 5O

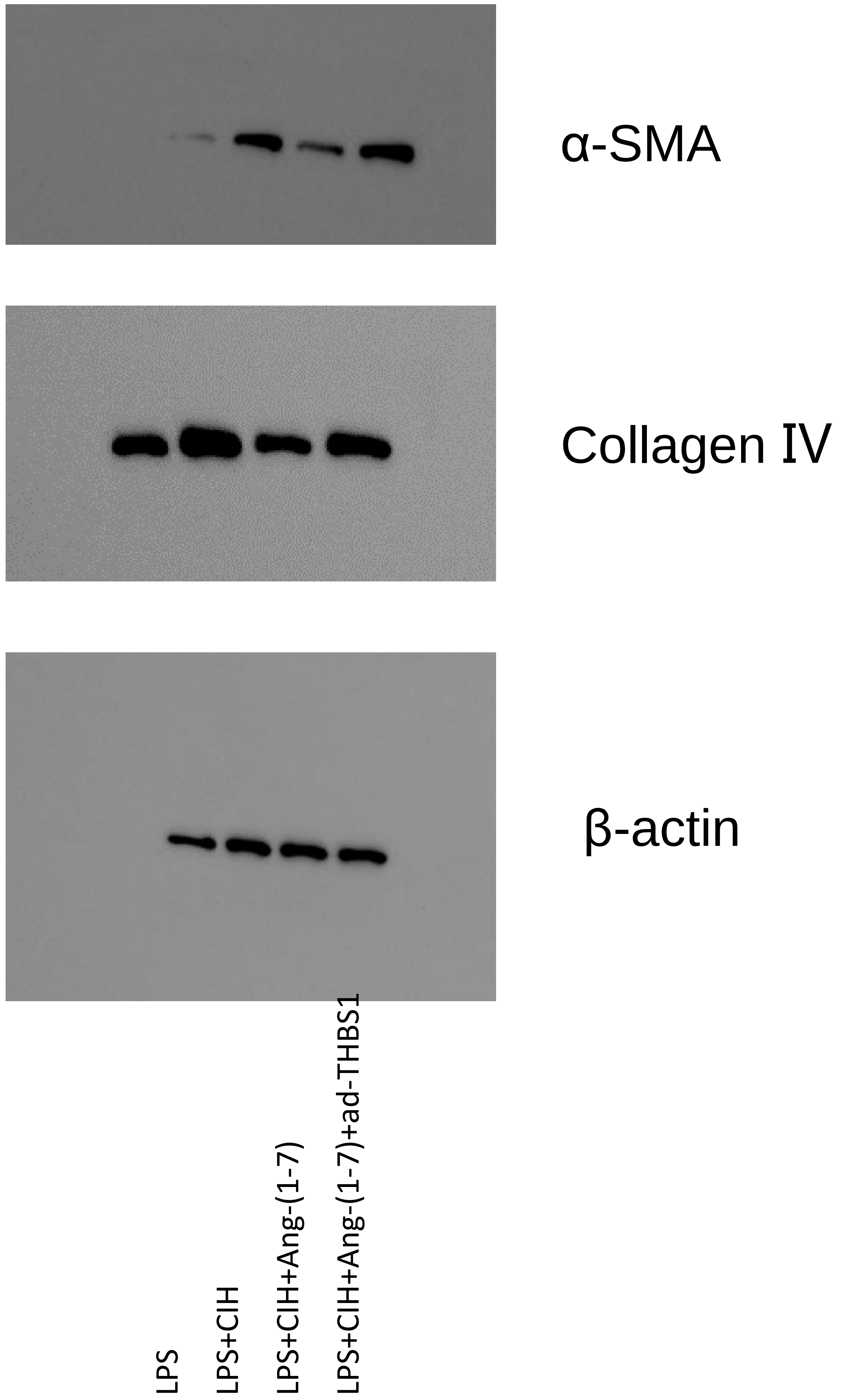

Figure 5

Figure 6C

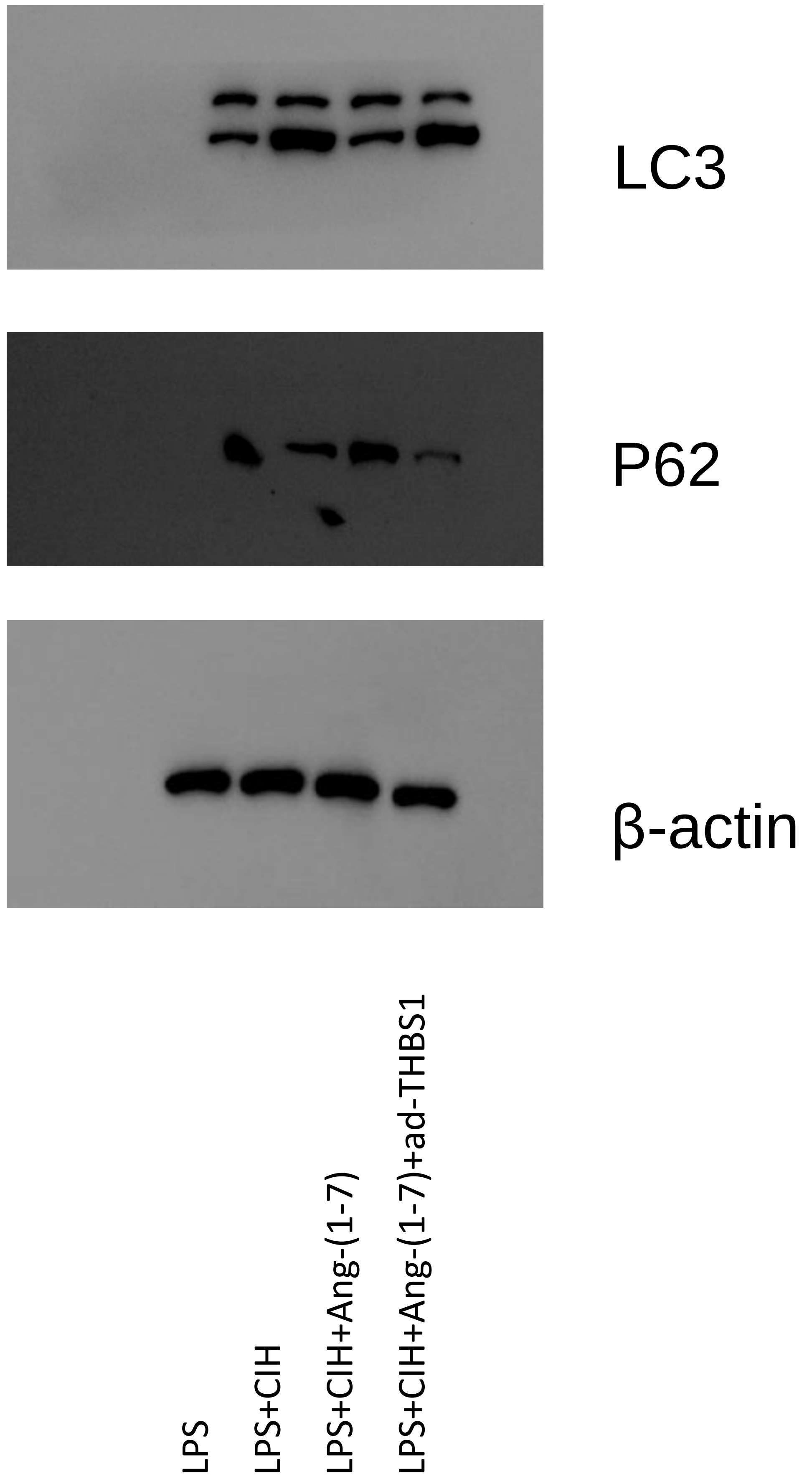

Figure 6

Figure 6D

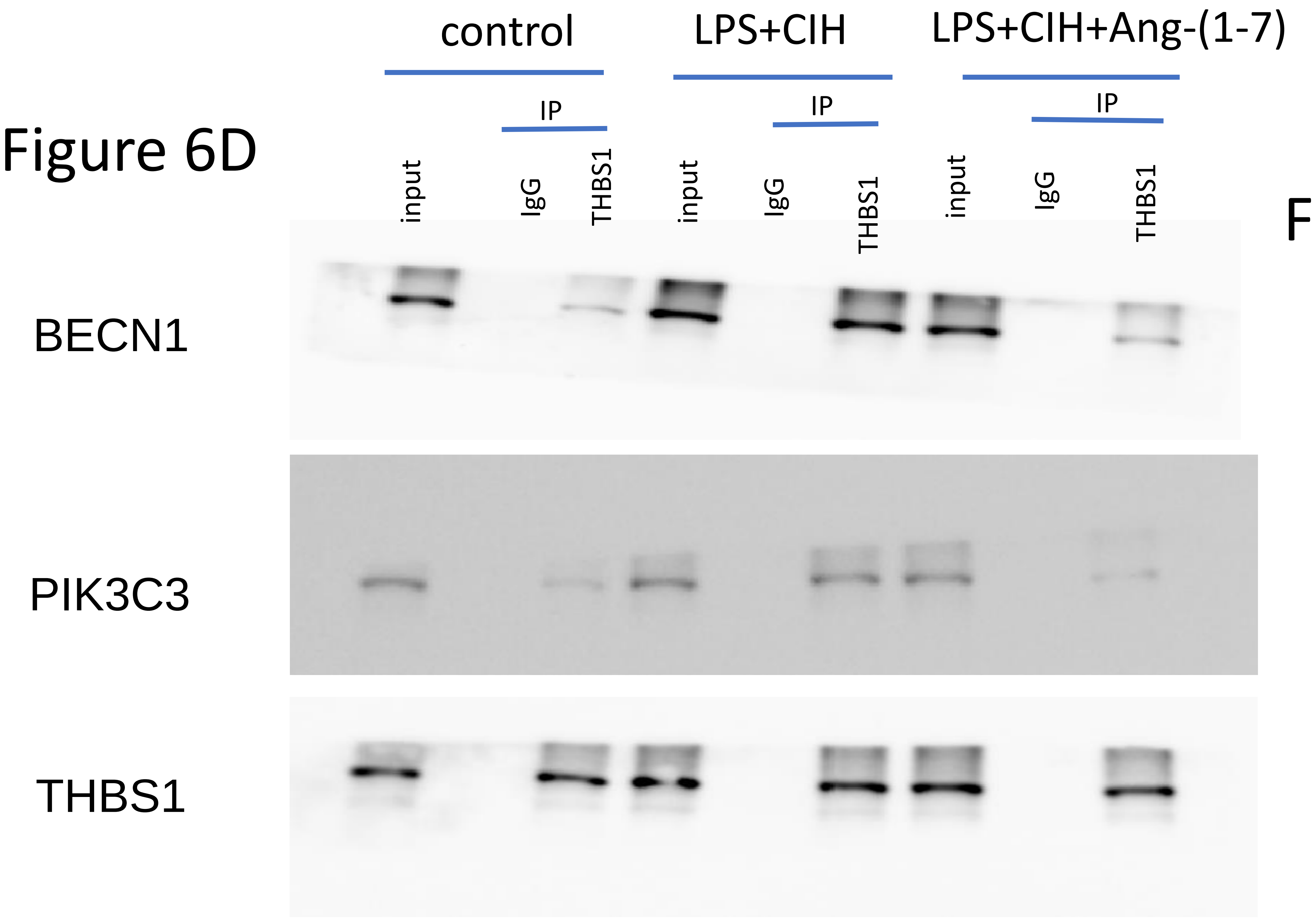

Figure 6E

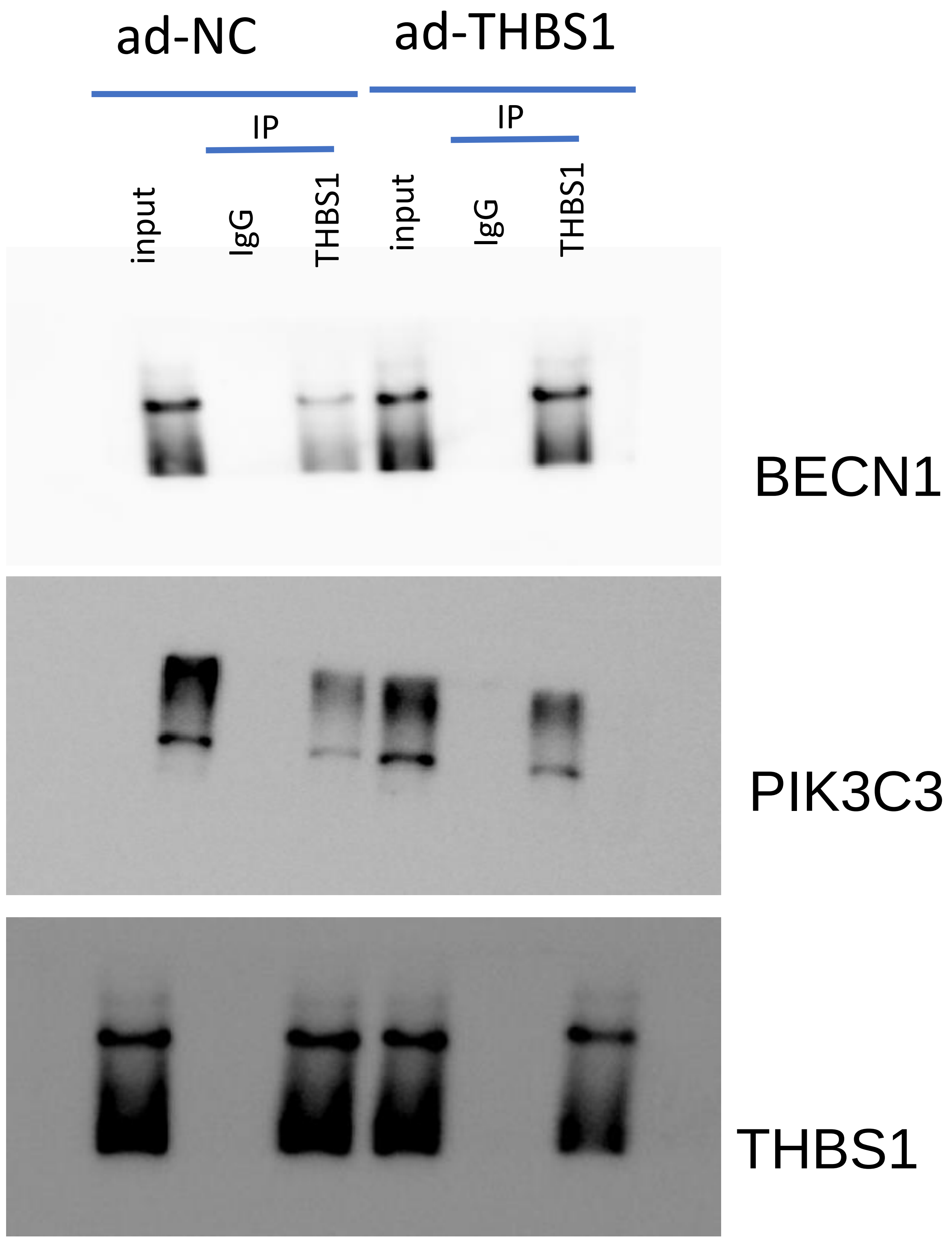

Figure 6G

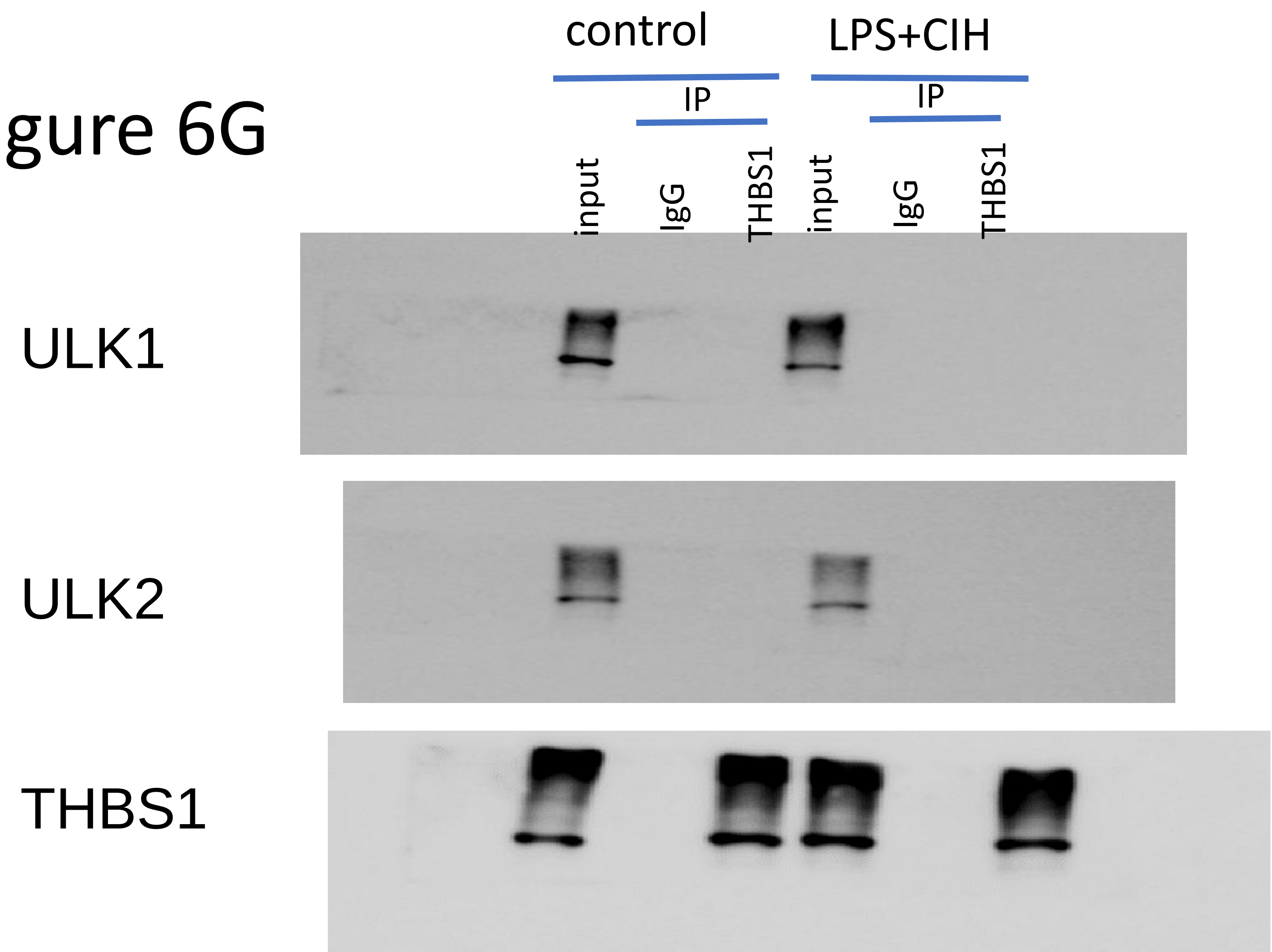

Figure 6

Figure 7F

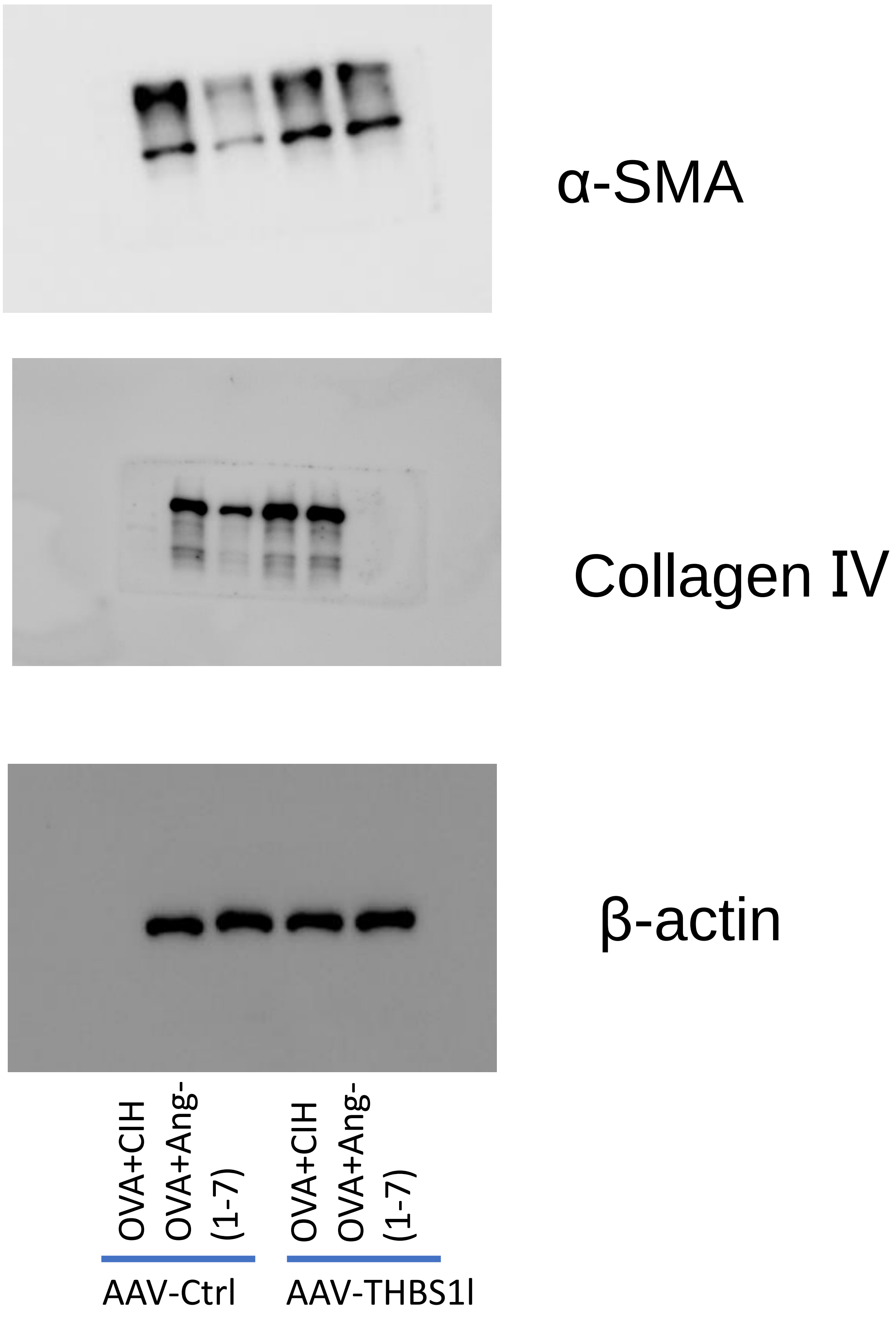

Figure 7G

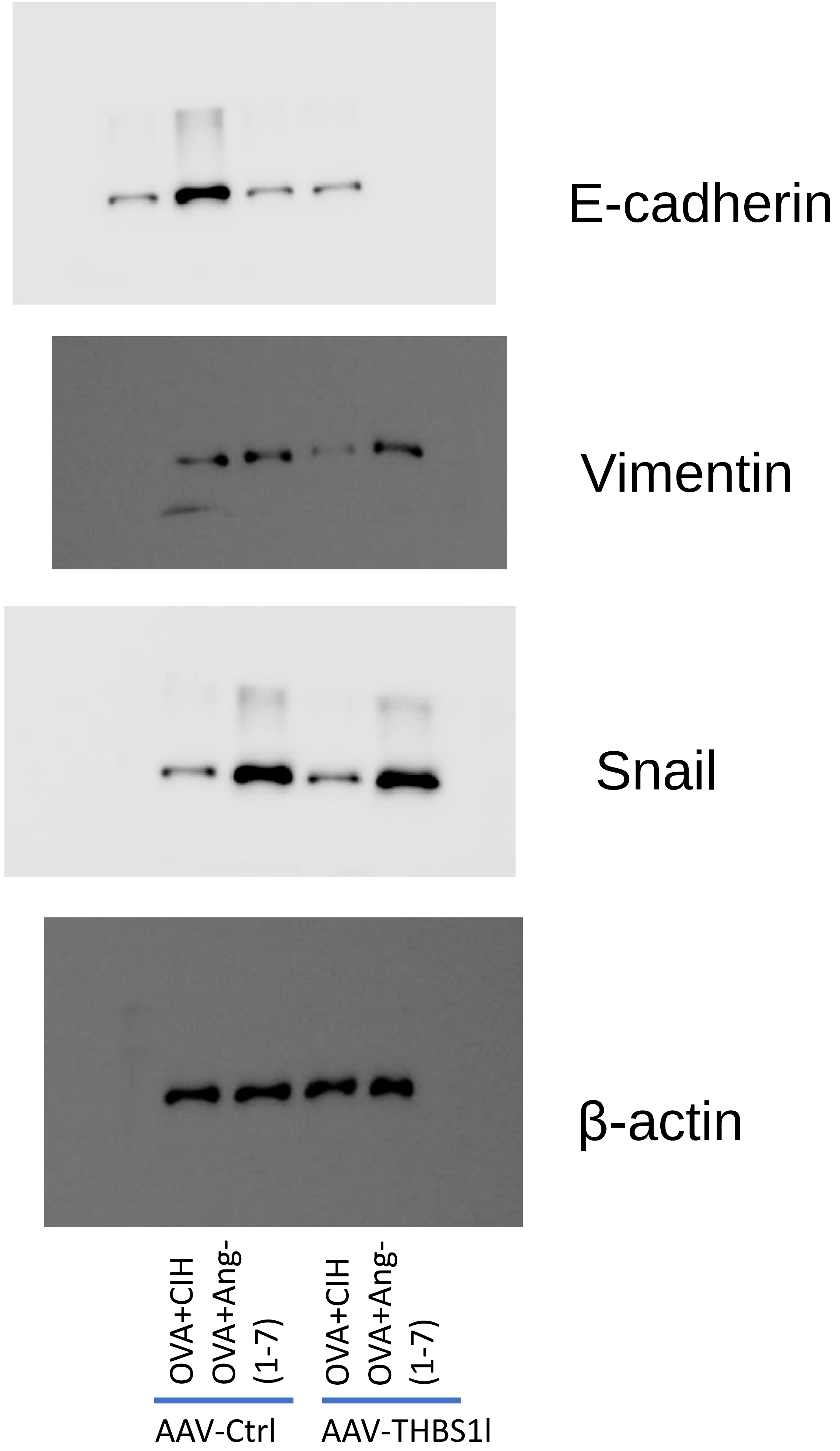

Figure 7I

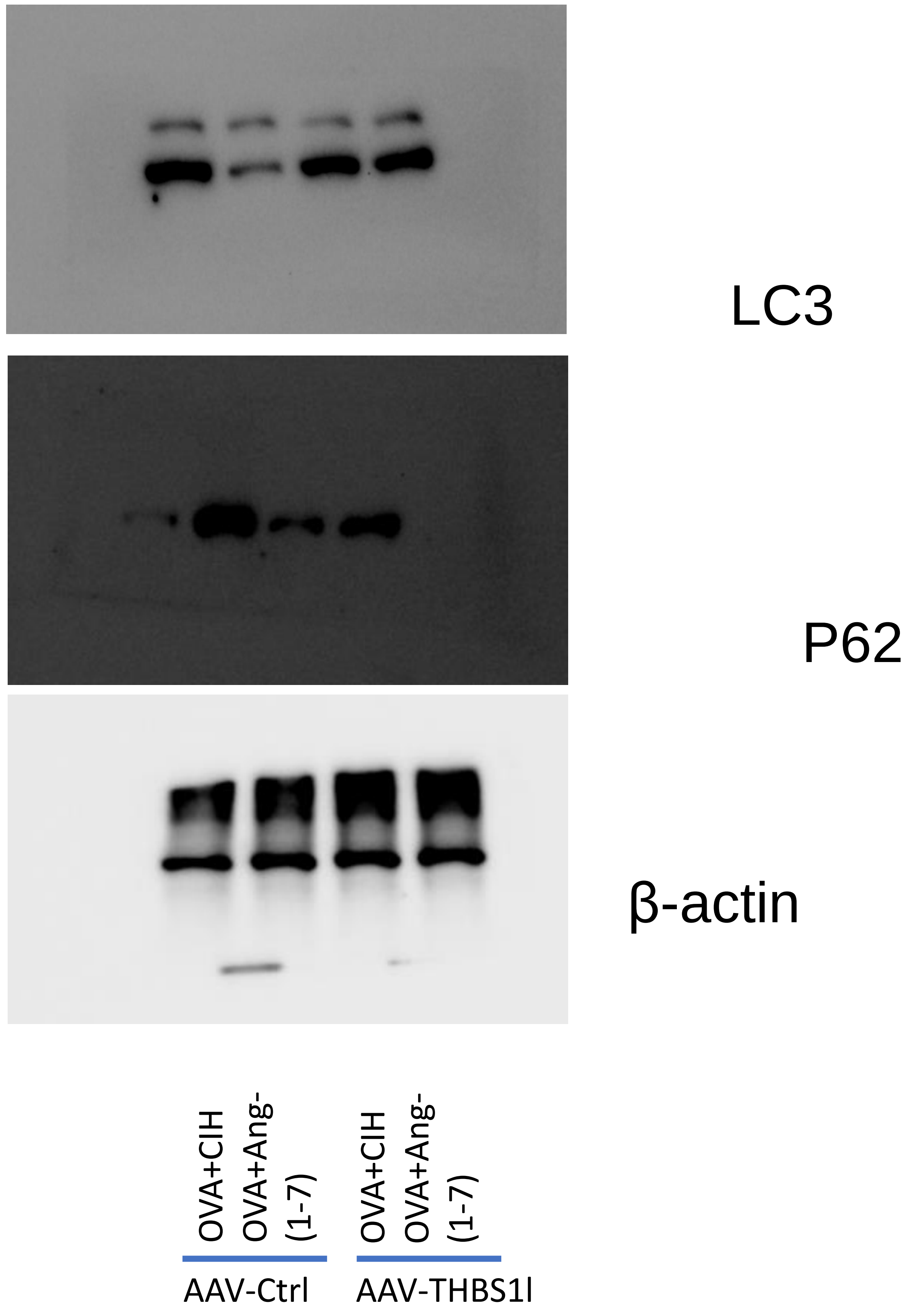

Figure 7

Figure S1G

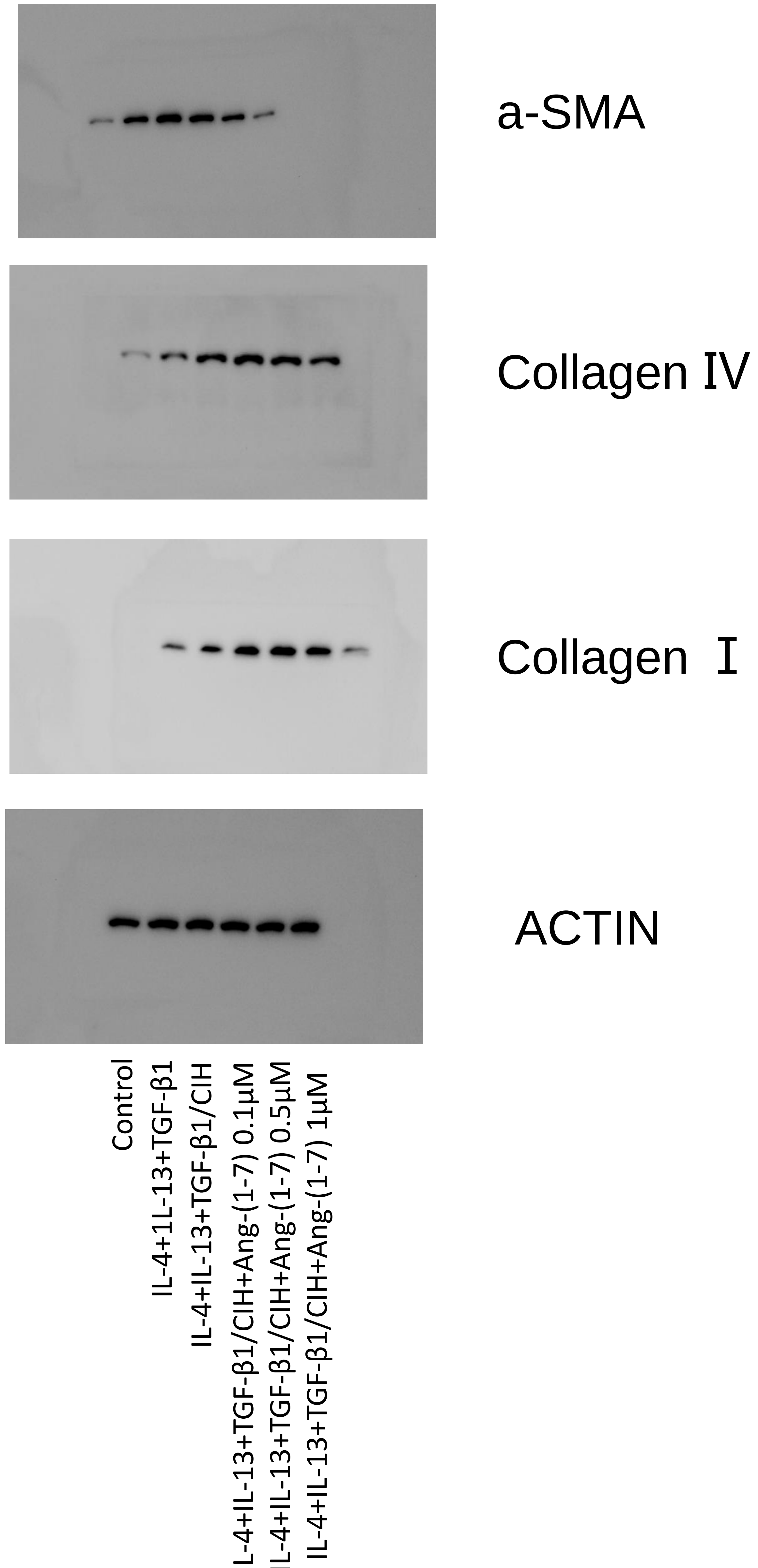

Figure S1I

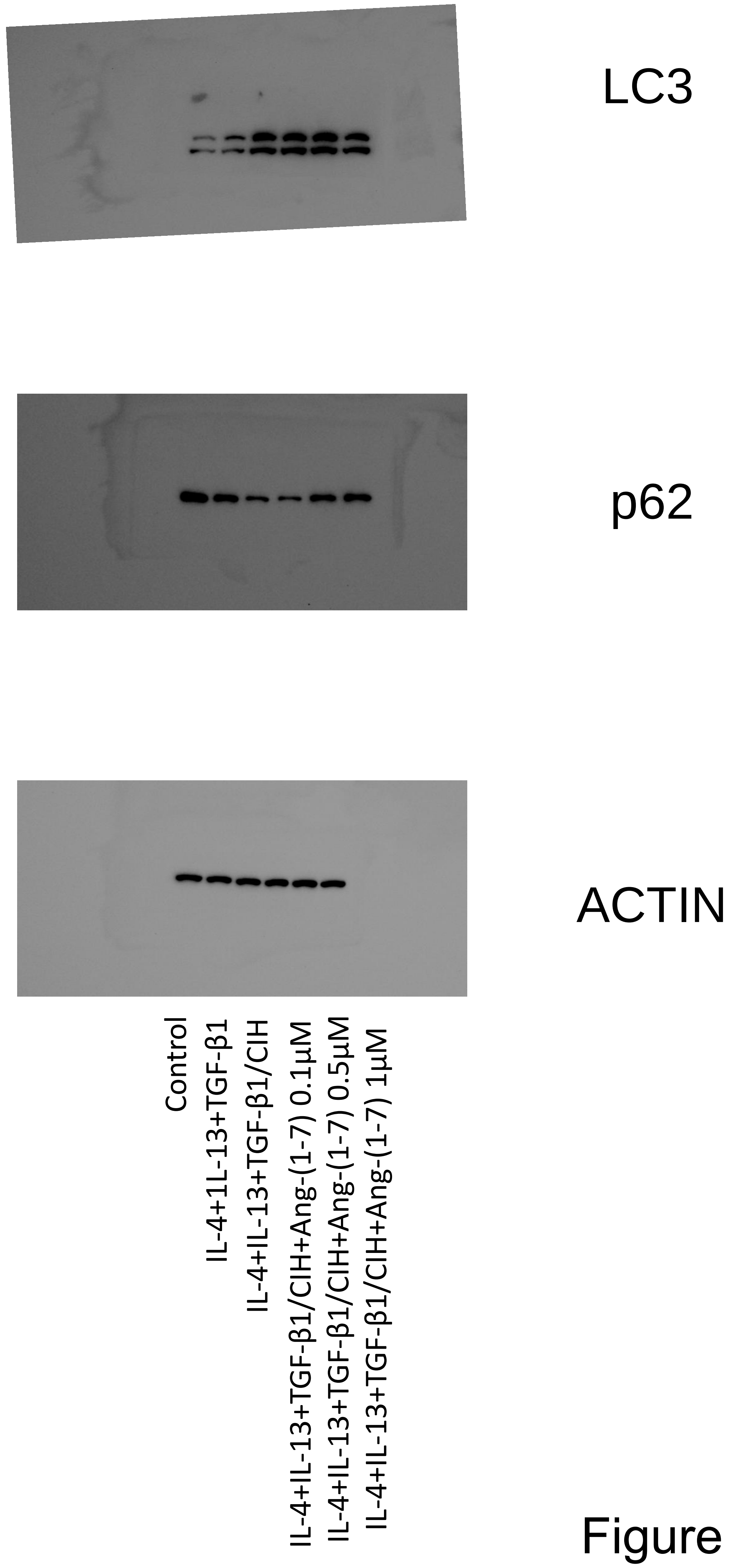

Figure S1

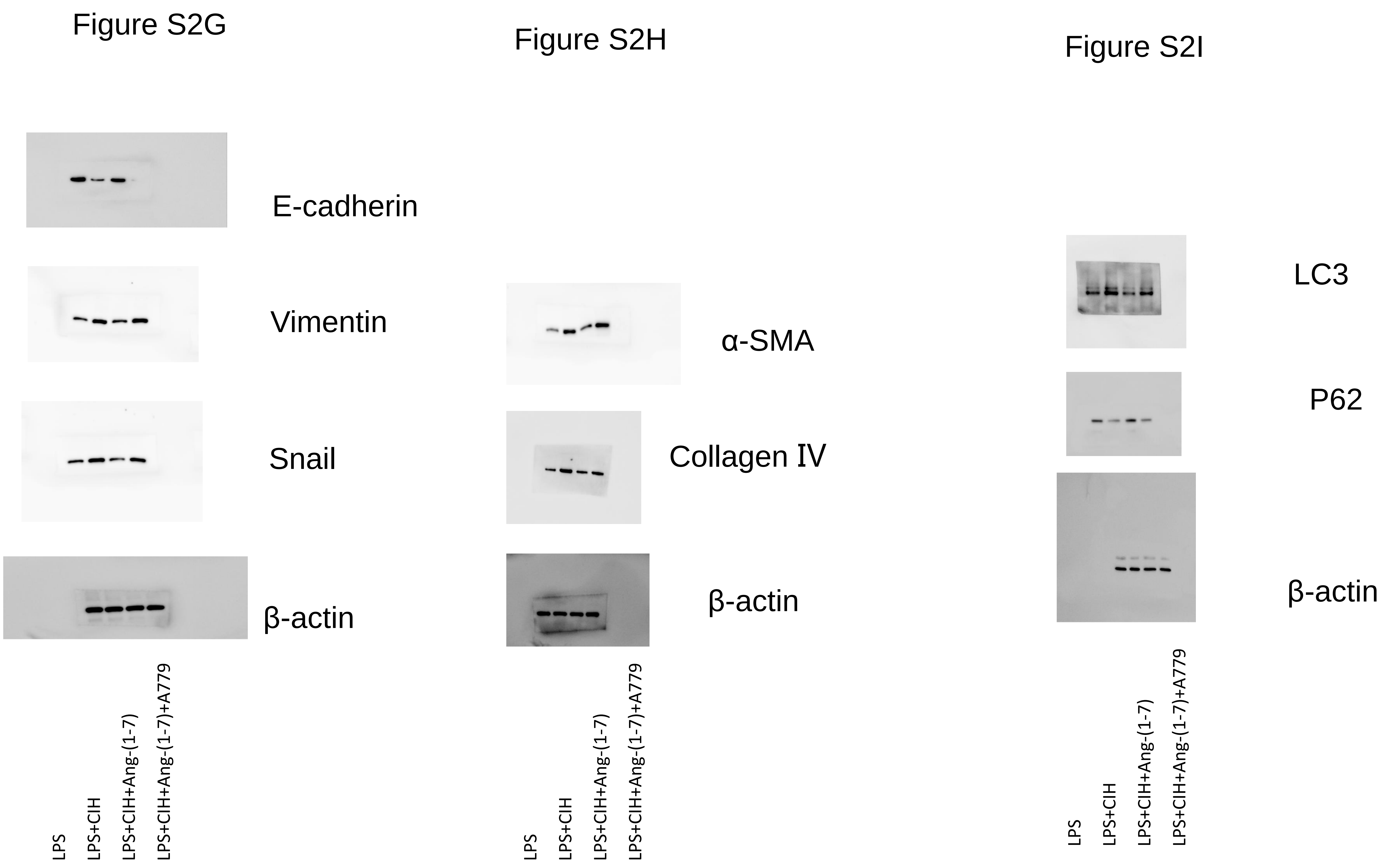
Figure S2G
E-cadherin
Vimentin
Snail
β-actin
LPS
LPS+CIH
LPS+CIH+Ang-(1-7)
LPS+CIH+Ang-(1-7)+A779
Figure S2H
α-SMA
Collagen IV
β-actin
LPS
LPS+CIH
LPS+CIH+Ang-(1-7)
LPS+CIH+Ang-(1-7)+A779
Figure S2I
LC3
P62
β-actin
LPS
LPS+CIH
LPS+CIH+Ang-(1-7)
LPS+CIH+Ang-(1-7)+A779

Figure S2
