# Supplementary material for: Exogenous Ang-(1-7) inhibits autophagy via HIF-1α/THBS1/BECN1 axis to alleviate chronic intermittent hypoxia-enhanced airway remodelling of asthma
Source: Cell Death Discov. 2023 Oct 2;9:366. doi: 10.1038/s41420-023-01662-0 (PMC10545676; doi:10.1038/s41420-023-01662-0)
Supplement: Supplementary file 6 — author-contribution-form [file 41420_2023_1662_MOESM6_ESM.pdf]

DECLARATION OF CONTRIBUTIONS TO ARTICLE

ADMC

Manuscript Number: CDDISCOVERY-23-1107R1

Journal Name: *Cell Death & Discovery* (the 'Journal')

Proposed Title of the Contribution:

Exogenous Ang-(1-7) Inhibits Autophagy via HIF-1α/THBS1/BECN1 Axis to Alleviate Chronic Intermittent Hypoxia-enhanced Airway Remodelling of Asthma (the 'Contribution)

Author(s):

Jian Ping Zhou, Yi Wang, Shi Qi Li, Jia Qi Zhang, Ying Ni Lin, Xian Wen Sun, Li Na Zhou, Liu Zhang, Fang Ying Lu, Yong Jie Ding, Qing Yun Li (the 'Authors')

For all *CDD* articles, each person named as an author in the published version must be able to show he or she has contributed substantially to the article.

Authorship credit should be based on 1) substantial contributions to conception and design, acquisition of data, or analysis and interpretation of data; 2) drafting the article or revising it critically for important intellectual content; and 3) final approval of the version to be published. Authors should meet conditions 1, 2 and 3.

Any person who cannot be shown to have made a substantial contribution to the article cannot be listed as an author in the final version. The name of any person who is deemed to have made a minor contribution can, however, appear in the Acknowledgments section of the article.

Please complete the table below to indicate the contributions of all named authors to the manuscript.

Author Full Name:	Specification of Contribution to the Manuscript:
Jian Ping Zhou	designed experiments,acquisition of data,and drafted the manuscript
Yi Wang	acquisition of data and help write the manuscript
Shi Qi Li	acquisition of data and help write the manuscript
Jia Qi Zhang	performed the additional experiment for manuscript revision
Ying Ni Lin	help write and revise the manuscript
Xian Wen Sun	help write and revise the manuscript
Li Na Zhou	help write and revise the manuscript
Liu Zhang	provided technical and methodological support
Fang Ying Lu	provided technical and methodological support
Yong Jie Ding	help write and revise the manuscript
Qing Yun Li	conception and revision of the manuscript
	All authors read and approved the final version of the manuscript.

Please complete the table below to indicate the contributions of all named authors to the figures.

Figure 1:

ZJP generated the H&E, Masson trichrome stain and ELISA data. ZJP assembled the figure.

Figure 2:

WY generated the immunofluorescence data. ZJP generated the western blot data. ZJP assembled the figure.

Figure 3:

WY generated the immunofluorescence data. ZJP generated the western blot data. ZJP assembled the figure.

Figure 4:

WY generated the immunofluorescence data. ZJP generated the western blot data. ZJP assembled the figure.

Figure 5:

LSQ completed the bioinformation analysis and generated the data of PCR and CHIP. WY generated the immunofluorescence data. ZJP generated the western blot data. ZJP assembled the figure.

Figure 6:

WY generated the immunofluorescence data and proximity ligation assay data. LSQ completed the COIP. ZJP generated the western blot data. ZJP assembled the figure.

Figure 7
ZJP generated the H&E, Masson trichrome stain and western blot data. WY generated the immunofluorescence data. ZJP assembled the figure.

Figure S1
ZJQ generated western blot and immunofluorescence data and ZJP assembled the figure.

figure s2

WY generated the immunofluorescence data. ZJP generated the western blot data. ZJP assembled the figure.

Signed for and on behalf of the Author(s):	Print Name:	Date:
	Qing Yun Li	2023-8-30
